# Prognostic and therapeutic TILs of cervical cancer—Current advances and future perspectives

**DOI:** 10.1016/j.omto.2021.07.006

**Published:** 2021-07-21

**Authors:** Ying Tang, Anne X.J. Zhang, Guangyu Chen, Yanheng Wu, Wenyi Gu

**Affiliations:** 1 Institute of Tumor, Guangzhou University of Chinese Medicine, Guangzhou, China; 2 Gillion ITM Research Institute, Guangzhou Hongkeyuan, Guangzhou, China; 3 Australian Institute of Bioengineering and Nanotechnology, The University of Queensland, Brisbane, QLD 4072, Australia

**Keywords:** cervical cancer, tumor-infiltrating lymphocytes, TILs, cancer immunotherapy, human papillomavirus, adaptive cell therapy, tumor prognosis, CD8^+^ T cells, CD4^+^ T cells, regulatory T cells

## Abstract

Cervical cancer is a top lethal cancer for women worldwide. Although screening and vaccination programs are available in many countries, resulting in the decline of new cases, this is not true for developing countries where there are many new cases and related deaths. Cancer immunotherapy through adaptive cell therapy (ACT) has been applied in clinics, but now much attention is focused on autogenic tumor-infiltrating lymphocyte (TIL)-based therapy, which has shown more specificity and better ability to inhibit tumor growth. Data from melanoma and cervical cancers confirm that tumor-specific T cells in TILs can be expanded for more specific and effective ACT. Moreover, TILs are derived from individual patients and are ready to home back to kill tumor cells after patient infusion, aligning well with personalized and precision medicine. In addition to therapy, TIL cell types and numbers are good indicators of host immune response to the tumor, and thus they have significant values in prognosis. Because of the special relationship with human papillomavirus (HPV) infection, cervical cancer has some specialties in TIL-based prognosis and therapy. In this review, we summarize the recent advances in the prognostic significance of TILs and TIL-based therapy for cervical cancer and discuss related perspectives.

## Graphical abstract

**Figure:**
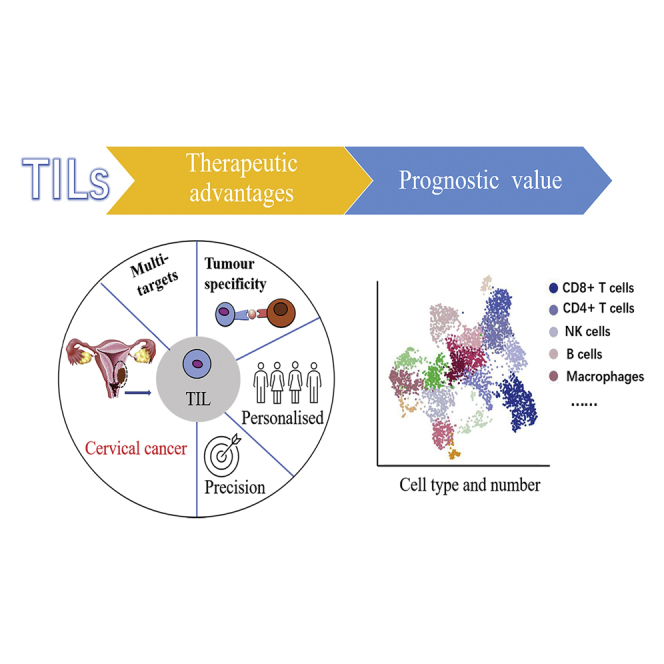

## Teaser

Tumor-infiltrating lymphocytes (TILs) of cervical cancer can be isolated and expanded as a personalized and precision medicine to specifically treat heterogeneous (multi-target) tumor cells of the patient. Furthermore, the phenotype and number of TILs or infiltrating immune cells (such as macrophages) have a significant value in predicting cancer progression and prognosis.

## Introduction

Despite the prevention and treatment options available for cervical cancer patients, such as human papillomavirus (HPV) screening, prophylactic HPV vaccines, surgery, radiotherapy, and chemotherapy, the disease burden remains high worldwide. Even though prophylactic vaccines, including bivalent, quadrivalent, and a new nine-valent vaccines, are commercially available and effective in protecting against >90% of HPV infection, they provide limited benefits to eliminate pre-existing infections.^1^ In fact, in 2018, there were still ∼569,000 new cases of cervical cancer diagnosed worldwide and ∼311,000 deaths reported that were attributed to cervical cancer. Of these, between 84% and 90% occurred in low- and middle-income countries such as South Africa, India, China, and Brazil.^2^ It is well known that the major cause of cervical cancer is persistent infection of the sexually transmitted HPVs, especially the high-risk types. Advanced cervical cancers can only be treated with chemotherapy or radiotherapy, but the outcomes are poor, with a median survival rate of only 16.8 months.^3^ Clearly, for these patients, alternatives and more effective treatments are urgently needed.

Cancer immunotherapy is emerging as a promising new approach to treat various cancers, such as chimeric antigen receptor T (CAR-T) cells^4^^,^^5^ and negative immune regulation-based therapies (especially after the Nobel prize announcement in 2018).^6^^,^^7^ These approaches aim to enhance host immunity or downregulate the negative regulators of the immune system to restore host immunity against cancers. Exciting achievements and outcomes were obtained in treating hematologic malignancies with CAR-T cells^4^^,^^5^^,^^8^ and advanced or metastatic solid tumors with antibodies to cytotoxic T lymphocyte antigen-4 (CTLA-4 and programmed death-1 (PD-1)/programmed death-ligand 1 (PD-L1).^9^, ^10^, ^11^, ^12^ Additionally, adaptive cell therapy (ACT) has been widely accepted and applied in clinics with some promising results,^13^ although it faces challenges of specificity and efficacy.^5^^,^^6^^,^^14^ As such, researchers were looking for more specific immune cells for ACT. In the late 1980s, researchers realized that tumor-infiltrating lymphocytes (TILs) are special immune cells and have a better ability to inhibit tumor growth.^15^^,^^16^ Recent data from melanoma and breast and cervical cancers further confirm that TILs contain more tumor-specific immune cells that can be expanded *ex vivo* for more effective ACT. The TIL-based therapy is expected to be more acceptable by clinicians and patients and more efficient than conventional ACT.^17^ In addition, it aligns well with personalized and precision medicine, as TILs are derived from individual patients and thus encompass more tumor-specific T cell populations of the patient^18^, ^19^, ^20^ and readily home back to tumor sites to kill the tumor cells. Therefore, TIL-based immunotherapy has advantages over conventional ACT, and it will offer a life-saving alternative treatment for cancer patients, including for cervical cancer.

There are some exciting advances in research and application of prognostic and therapeutic TILs in cervical cancer, although relatively fewer data are available on TIL therapy compared to other cancers such as melanoma and breast cancer. In cervical cancer, many studies are focused on the prognostic significance of TILs, their types, numbers, and functionality. For TIL-based therapy, recent studies have moved toward the selection of HPV-specific TILs and are exploring methods to enhance the immune functions of TILs. There are some specific characteristic features of TIL therapy for cervical cancer due to its association with HPV infection and viral oncogenes in tumor development. It is thus necessary to summarize the current advances in the research and application of TILs for cervical cancer to provide a guide toward future development of TIL therapy for this cancer. In this review, we first introduce the general concept and mechanisms of TILs and TIL-based therapy, followed by more specific descriptions of prognostic values of each TIL population. We then summarize the TIL-based therapy for cervical cancer and highlight the reported methods for TIL enhancement. Finally, we discuss the future perspectives of TIL studies and TIL therapies for cervical cancer.

## TILs and TIL-based immunotherapy

### History and concept

TILs were first described in 1863 by the German pathologist Rudolf Virchow as white blood cells present in malignant tumors.^21^ Initially, TILs were considered to constitute the origin of cancer in chronic inflammation sites.^21^ Later, researchers debated on whether these lymphocytes provided a favorable environment for cancer growth.^22^ The relationship between the degree of immune cell infiltration and prognosis was first discovered in breast cancer cases in 1949.^23^ In 1969, Clark et al.^24^ described the lymphocyte infiltration of primary cutaneous melanoma, and Day et al.^25^ and Tuthill et al.^26^ later found that these infiltrating lymphocytes had prognostic significance. Animal studies have confirmed that lymphocytes of immune donors can be transferred to tumor recipients to mediate tumor recurrence.^27^, ^28^, ^29^ On this basis, Donohue et al.^30^ further proved that the simultaneous administration of interleukin (IL)-2 *in vivo* can enhance the anti-tumor efficacy. However, due to the lack of the source of syngeneic anti-tumor lymphocytes, the translation of this work in humans was inherently postponed.

In 1986, Rosenberg et al.^16^ at the National Institutes of Health (NIH) first used TILs from mice to demonstrate that the combined use of autologous TILs and cyclophosphamide can induce metastatic tumor shrinkage. Subsequently, they published the first landmark human research in 1988, in which they showed that TILs could induce cancer regression in patients with metastatic melanoma.^15^ From 1987 to 1992, they analyzed 86 melanoma patients who were treated with TILs followed by high-dose IL-2 and showed a 34% overall responsive rate (ORR), with and without prior IL-2 exposure. Five patients (6%) had complete remission (CR), but only two patients lasted 21 and 46 months.^31^ Since then, the NIH and other institutions have made considerable efforts to improve the therapy by modifying the protocol of generating and selecting TILs and changing the pre-TIL treatment plan. In 2010, Dudley et al.^32^ developed “young TILs,” which shortened the growth time of TILs. In 2019, the US Food and Drug Administration (FDA) granted TIL treatment LN-145 as a breakthrough treatment plan.^33^^,^^34^ This is the first time that cell immunotherapy for solid tumors received this award, and it is thought that it is only a matter of time before this treatment is launched onto the market. At the 2020 American Association for Cancer Research meeting, the Moffitt Cancer Center in the US reported that the TIL treatment of PD-1-resistant non-small cell lung cancer (NSCLC) patients showed positive results with durable tumor remission, indicating a great hope for lung cancer treatment.

### TILs represent host immunity against cancer: The prognostic value

The *in situ* presentence of TILs (type and number) in tumors is a good reflection of the dynamic interaction of the host immune system to tumor antigens and the tumor microenvironment (TME). Indeed, the analysis of TIL type, number, and ratio (phenotype changes) can provide useful information for tumor progression and the prognosis of treatment. In general, cervical cancer TILs consist of several major subtypes of immune cells, and each subtype relates to its unique function. The subtypes include CD4^+^ and CD8^+^ αβ T, γδ T, B, natural killer (NK), and regulatory T cells (Tregs). In some reports, TILs are also referred to as tumor-infiltrating immune cells, which include macrophages. Therefore, TILs represent a heterogeneous population of immune cells present in the tumor tissues. These immune cells clearly cannot effectively eradicate the tumor when detecting TILs. The reasons are complicated. One reason may relate to insufficient anti-tumor cells, or they have been senescent or allergic, and the presence of immunosuppressive cells such as Tregs.^35^ Another reason, which is more clear at this time, concerns the overexpression of negative regulators and the inhibitory TME.^36^

However, more and more studies have shown that the degree and composition of the host’s immune response to tumors have prognostic and predictive significance for many solid malignant tumors. Evaluating TILs in tumors is becoming increasingly important in the search for the ideal biomarker to select patients with the highest likelihood of responding to an immunotherapeutic. Therefore, TIL evaluation is recommended as a biomarker for routine histopathology reports in some cancer types,^37^ although not yet in cervical cancer.

### TIL-based immunotherapy

Although it was thought that TILs present in the tumor or TME were due to inflammation, it has been proved by several studies that TILs consist of a larger portion of tumor-specific T cells, including CD8^+^ CTLs and CD4^+^ T helper (Th) cells. This lays the foundation of TIL-based immunotherapy (TIL therapy). In general, TILs from tumor tissues have a percentage around 60% of tumor-specific T cells with a strong tumor killing ability, while most ACTs use immune cells from blood with a much lower percentage of tumor-specific T cells (e.g., > 0.5%). Moreover, TILs contain more tumor-specific CTL populations that can react to specific mutations (neoantigens) in tumor of the individual patient and thus have a wider spectrum of killing tumor cells, which will finally increase the specificity and cytotoxicity of personalized TIL therapy. From these aspects, TIL therapy has advantages over other cell-based immunotherapies.

**Figure 1:**
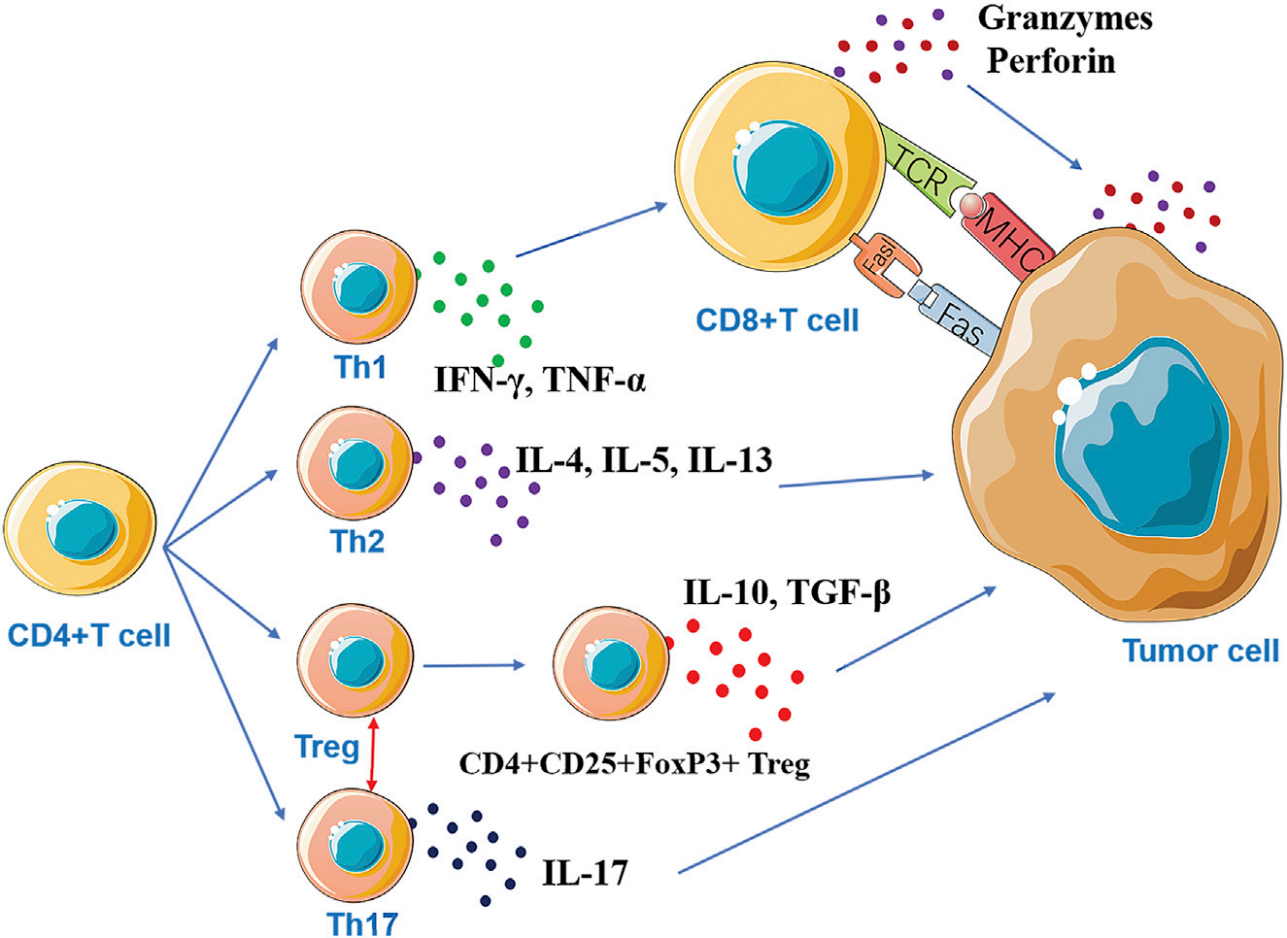
Schematic illustration of immune functions of CD4^+^ and CD8^+^ TILs CD4^+^ T cells are plastic and can differentiate to several subtypes that produce different cytokines and exhibit unique functions to interact with CD8^+^ and tumor cells, whereas CD8^+^ T cells mainly perform the cytotoxicity to tumor cells via perforin/granzyme or induce apoptosis via the Fas/FasL pathway.

The CD8^+^ CTLs in TILs can recognize antigen epitopes composed of 8- to 10-aa polypeptides presented by major histocompatibility complex (MHC) class I (human leukocyte antigen [HLA]-A, -B, -C) and kill tumor cells in a specific manner, whereas CD4^+^ T cells recognize at least 13-aa peptides presented by MHC class II (HLA-DR, -DP, -DQ) molecules. MHC class I proteins are basically expressed in all nucleated cells and are therefore widely distributed, including tumor cells. However, they may disappear during tumor progression.^38^ In contrast, MHC class II proteins are often expressed on antigen-presenting cells (APCs); however, they may be abnormally expressed on tumor cells due to the inflammatory environment around the tumor or tumor transformation.^39^^,^^40^ For example, MHC class II molecules are expressed on the surface of many melanoma cells, and interferon γ (IFN-γ) can induce MHC class II expression.^41^^,^^42^ CD8^+^ TILs can readily home to the tumor and can kill tumor cells directly and indirectly. Directly, they can recognize tumor antigens and secrete factors, such as perforin and granzyme, to cause tumor cell death. Indirectly, they can activate the Fas-FasL pathway to induce apoptosis of tumor cells.^43^^,^^44^ Memory T cells can be classified into tissue-resident memory T (TRM) cells and central memory T (TCM) cells.^45^^,^^46^ Under the stimulation of IL-15 and transforming growth factor β (TGF-β), CD8^+^ T cells can differentiate into TRM cells with high expression of CD69 and granzyme B.^47^^,^^48^ CD4^+^ T cells can differentiate into Th1, Th2, Th17, and Tregs, and they play a regulatory role in the immune response.^48^ IFN-γ secreted by Th1 cells can cause the activation of cytotoxic CD8^+^ T cells, dendritic cells (DCs), and macrophages, producing effective anti-tumor effects; Th2 cells mainly secrete IL-4, which can lead to the activation of tumor-promoting macrophages to promote tumor growth. CD4^+^ T cells also have a strong plasticity and can differentiate into different cell phenotypes. For example, Th17 cells can be transformed into Th1 and Th2 phenotypes.^49^ Downs-Canner et al.^50^ found that Th17 cells can be transformed into tumor-induced FoxP3^+^CD4^+^ T cells (Figure 1).

The principle of TIL therapy is to enhance and restore anti-tumor immunity of TILs through transferring the cells from the immunosuppressed TME to a promoting environment to expand *in vitro* and reach a high enough number for patient infusion. Therefore, an important goal of TIL studies is to determine the subtype composition in the initial TIL population and preferentially expand them. Early animal and clinical studies have shown that TILs at an early stage of differentiation with longer telomeres and a central memory phenotype (CD62L^+^, CD27^+^, and CD28^+^) are associated with longer persistence and a better clinical response in patients.^51^, ^52^, ^53^ In 2010, Dudley et al.^32^ developed the young TIL method by pooling lymphocytes from multiple tumor sites to obtain the cell number required for rapid expansion. By shortening the culture time, TILs with high expression of CD27 and CD28 and longer telomeres are enriched.^54^ This faster expansion method also brings significant clinical benefit, reducing the number of patients who do not meet the treatment conditions due to rapid disease progression and clinical decline during TIL production. Weber and the Moffitt Cancer Center enhanced TIL production by targeting 4-1BB, which is a costimulatory molecule on activated T cells, involved in T cell proliferation and antigen-specific cytolytic activity.^55^ Another area of concern is whether other T cell cytokines, such as IL-15 and IL-21, should be used instead of IL-2 alone during the production of TILs. Unlike IL-2, which promotes effector T cell differentiation and Treg proliferation and supports T cell activation-induced cell death (AICD), IL-15 and IL-21 seem to induce a younger, less differentiated central memory phenotype and do not promote AICD.^56^^,^^57^

### Limitations of TIL therapy

Although TIL therapy has many anticipated benefits described above, it clearly has limitations. First, TIL therapy is a personalized immunotherapy; because infusion products must be produced according to the specific conditions of patients, this results in a relativity high cost. Second, the production time of TIL products is long, which is not conducive to the control of the disease for some patients with rapid cancer progression. In addition, a highly specialized good manufacturing practice (GMP) facility and well-trained personnel are required, which again is costly and time-consuming. Even though in the future with the development of computerized bioreactors that can at least partially replace some of the processing work of production and personnel, the process cannot be fully automated due to the collection of the original heterogeneous materials (tumor fragments or digests). Moreover, there is a need to clarify the role of high-dose IL-2 in TIL treatment. For example, after TIL infusion, low-dose IL-2 may be used, or even a new type of IL- 2, including IL-2 mutein substitutions at key sites or IL-2 analogs, which have been shown to reduce high-affinity IL-2 receptor binding,^58^ thereby reducing IL-2 toxic side effects, especially blood vessel expansion and pulmonary edema, and will not activate and expand CD4^+^Foxp3^+^ Tregs.^59^^,^^60^

## Prognostic TILs of cervical cancer: Cell type, number, and ratio

**Figure 2:**
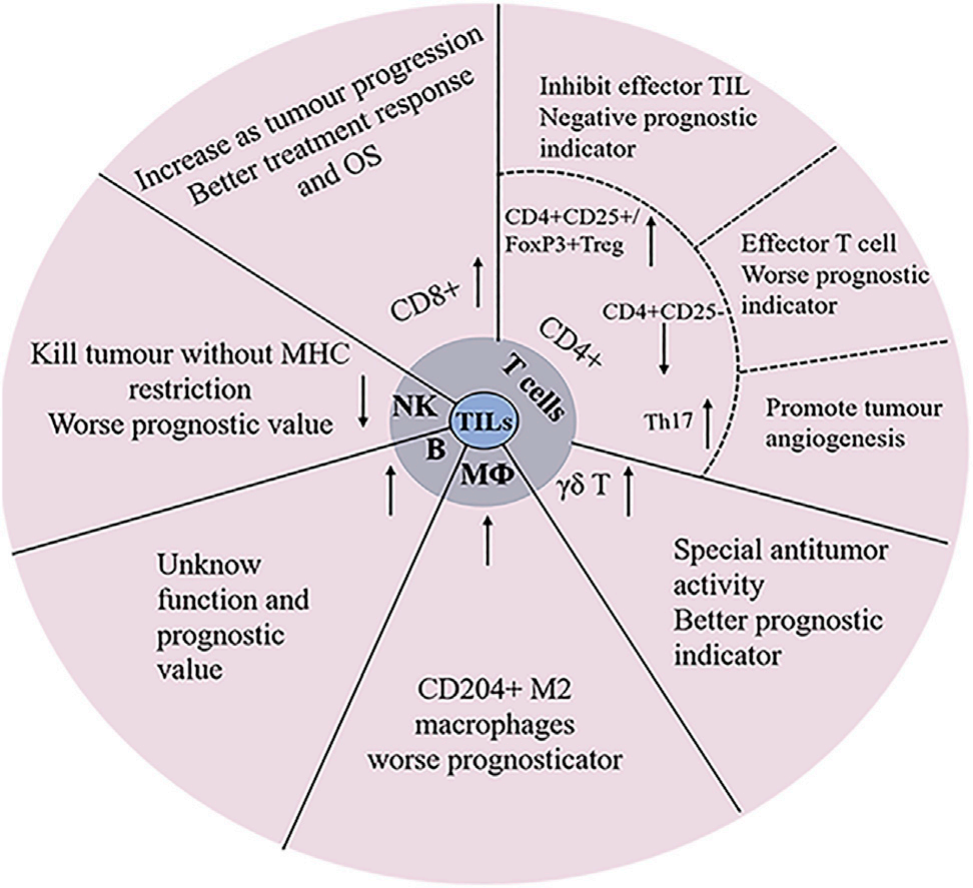
Subtypes and prognostic significance of TILs in cervical cancer Cervical cancer TILs mainly consist of CD8^+^, CD4^+^ T cells, NK cells, Tregs, B cells, and macrophages. Each has its unique function and own prognostic value.

In cervical cancer, most current studies focus on evaluating the prognostic values of TIL types and numbers *in situ* or *ex vivo*. Indeed, the number and composition of TILs are a good reflection of the process of the host immune system interacting with tumor cells and the TME, thus providing an indication for cancer progress and treatment outcome. These TILs mainly consist of CD8^+^ and CD4^+^ T cells, NK cells, Tregs, and Th17 and γδ T cells. Some studies also included B cells and macrophages. These cells are functionally different and thus are reviewed separately with regard to their prognostic values in cancer progression and treatment outcomes (Figure 2).

### Effector TILs of CD8^+^ and CD4^+^ T cells

#### Association with cancer progression

Generally, CD8^+^ and CD4^+^ cells are the predominant cell types in TILs, and their presentence is a positive prognostic indicator for a better outcome of the patients. CD8^+^ cells, the major population of CTLs, are responsible for killing tumor cells in an MHC class I-matched manner, while CD4^+^ cells mainly provide help to CD8^+^ cells. Because they both assume primary immunity to tumor cells and are the most studied TILs in cervical cancer, they are reviewed together. A study analyzed the location and quantity of CD8^+^ and CD4^+^ TILs in the cervical neoplastic milieu of 72 samples using immunohistochemistry (IHC). The patients included four cohorts: 23 HPV non-infected (HPV^−^) normal cervix, 20 HPV-infected (HPV^+^) normal cervix, 17 HPV^+^ low-grade cervical intraepithelial neoplasia (CIN), and 12 HPV^+^ high-grade CIN. The results showed that a low level of TILs in normal cervix and a high level of TILs in CIN were present with a trend of TIL levels increasing with increases of the grade of CIN (p < 0.0001).^61^ In detailed analysis, they found that in the epithelial layer, CD8^+^ TILs were significantly higher than CD4^+^ TILs in HPV^+^ normal cervix, while the trend decreased with increasing grade of CIN (p = 0.011). In the stromal layer, CD4^+^ TILs were predominant in all groups, but no statistical difference was found between these groups. However, CD8^+^ TIL levels were significantly higher than those in CD4^+^ TILs in severe dysplastic cases,^61^ which is further supported by a similar study from 96 tissue section samples (including 26 CIN1, 21 CIN2, 25 CIN3, and 24 squamous cell carcinoma [SCC] samples).^62^ Because HPV mainly infects epithelial cells, these data suggest that CD8^+^ TILs are at the frontline, fighting virus-infected cells as well as cancer cells.

The cell density and distribution of CD3^+^, CD4^+^, and CD8^+^ TILs in paraffin-embedded tissues were also analyzed for their association with other clinicopathological features such as International Federation of Gynecology and Obstetrics (FIGO) stage, lymphovascular invasion (LVI) status, and lymph node metastasis of 96 cervical cancer patients. CD3^+^ (p = 0.010), CD4^+^ (p = 0.045), and CD8^+^ (p = 0.033) TILs in the central tumor area and CD8^+^ TILs in the invasive margin area (p = 0.004) showed significant higher levels in LVI(+) than in LVI(−) patients. When patients were grouped by status of lymph node metastasis, significant differences of FIGO stage (p = 0.005), LVI status (p = 0.003), and higher CD3^+^ TIL levels in the central tumor area (p = 0.045) were observed.^63^

Not only in the tissue section, but TILs directly cultured from biopsies of cervical cancer patients show a similar association. A study compared lymphocytes in cervical tissues from 19 patients with pathologically confirmed CIN and from 20 patients with normal cervices undergoing hysterectomy for benign indications. The percentage of CD4^+^ T cells was significantly depressed (p = 0.04) in dysplastic tissue as compared to normal cervical tissue. In contrast, the proportion of CD8^+^ T cells was significantly increased in the dysplastic tissue (p = 0.0001).^64^ All of the above data clearly indicate that the higher number and density of CD3^+^ and CD8^+^ TILs are positively associated with the progress of cervical cancer. A systematic review and meta-analysis on the prognostic significance of intra-tumoral CD3^+^ and CD8^+^ lymphocytes showed that both TILs had a positive effect on patient overall survival (OS) of cervical cancer with a hazard ratio (HR) of 0.58 and 0.71, respectively, for death,^65^ highlighting that CD3^+^ and CD8^+^ TILs have prognostic values not only for cancer progression but also patient OS.

Although CD3^+^ TILs include a CD4^+^ subset, the direct evidence of CD4^+^ T cell association with cancer progress is not strong. Instead, a few studies reported that the decreased cell number of CD4^+^ T cells was associated with worse long-term survival rates of cervical cancer patients. For example, a study retrospectively analyzed 40 biopsy samples from Chinese cervical cancer patients and found, when considering the deaths and surviving cases as separate groups, that the number of CD4^+^ T cells was significantly lower in patients who died compared with those who survived (26.33 ± 11.80 versus 47.79 ± 38.18, p = 0.023).^66^ Similarly, the proportion of CD4^+^ T cells was found to be significantly lower in tumors from patients (stage IA to IIA cervical carcinoma) with lymph node metastasis (n = 8) than in those from patients without lymph node metastasis (n = 22) (24.5 versus 32.7, p = 0.001). In addition, the proportion of CD4^+^ T cells was much lower in bulky tumors (n = 5) than in non-bulky tumors (n = 25) (21.4 versus 32.5, p < 0.001).^67^ These data collectively indicate that the decreased CD4^+^ TILs represent a worse prognostic sign for tumor progression and the chance of survival. Supporting this, an analysis of the composition of tumor-infiltrating immune cells (TICs) in cervical cancer based on RNA expression data with a metagene approach called CIBERSORT by Wang et al.^68^ showed that the difference of immune infiltration profiles between cervical cancer and normal tissues was the higher levels of activated memory CD4^+^ T cells present in cancer cases as an independent factor with favorable OS according to Cox regression analysis (HR = 0.71, 95% confidence interval [CI]: 0.57–0.89; p = 0.003).

#### Association with clinical treatment

Some studies evaluated the association of TILs with chemotherapy or radiotherapy (RT) in cervical cancer patients, showing that the abundant presence of CD8^+^ TILs indicates better treatment responses or outcomes of the patients. Martins et al.^69^ analyzed CD8^+^ TILs in 21 samples from patients with cervical SCC who were divided into responders (11/21) and non-responders (10/21) according to standard chemoradiation therapy response and found that responders showed an increase in numbers of CD8^+^ cells compared to non-responders. A pilot study also compared combinational treatment with cisplatin and paclitaxel and monotherapy in 13 primary cervical tumor patients, before and after neoadjuvant chemotherapy (NACT).^70^ Using multiplex IHC, the authors found that a significant decrease of proliferating Ki67^+^CD3^+^CD8^−^ T cells was observed in the tumor stroma after cisplatin and paclitaxel treatment, with increased rates of cytotoxic CD8^+^ T cells, including activated CD8^+^Tbet^+^ T cells. No such effect was observed on the number of TILs in the cervical TME after treatment with cisplatin only, suggesting that the combinational treatment attracted more TILs than did cisplatin monotherapy.

Radiation therapy could have direct cytotoxic effects on TILs, but it could also have immune stimulatory effects to increase immune cell infiltration. A study used a minimally invasive cervical cytobrushing method to analyze the kinetics of intra-tumoral immune cell changes in patients with cervical cancer during chemoradiation treatment (CRT). Cervical brushings were obtained from 20 patients with cervical cancer at baseline and during CRT. Matching peripheral blood mononuclear cells (PBMCs) were analyzed from nine patients at the same time points. The authors observed a significant decline in CD3^+^ total T cells, as well as in CD8^+^ and CD4^+^ T cell subsets, in the first week of treatment from baseline, followed by variable expansion at weeks 3 and 5. This coincided with higher levels of proliferating CD8^+^ T cells expressing Ki67 at week 3 of treatment. The percentages of activated CD8^+^ T cells expressing CD69 continuously increased during the course of treatment whereas these changes were not observed in PBMCs.^71^ These results indicated that CRT may be immune activating at the site of the tumor rather than in the peripheral blood. The results also show that the sequential analyses of the local TILs in the TME are more important for the prognosis of CRT.

In addition, a study reported the outcome of 71 patients with adenocarcinoma (AC) of the uterine cervix who underwent definitive radiotherapy comprising external beam radiotherapy and intracavitary brachytherapy with or without concurrent chemotherapy. Immunohistochemical detection of the expression of PD-L1 and CD8 showed that the 5-year locoregional control (LC), OS, and progression-free survival (PFS) rates for all patients were 61.8%, 49.7%, and 36.1%, respectively. Patients with CD8^+^ TILs in the tumor nests had significantly better OS than did patients without CD8^+^ TIL infiltration (5-year OS: 53.8% versus 23.8%, p = 0.038),^72^ suggesting that the presence of CD8^+^ TILs has the potential as an independent favorable prognostic factor for patients with adenocarcinoma of the uterine cervix after definitive radiotherapy.

### NK cells

NK cells are an important subset of TILs, and emerging evidence from other cancers indicates their high importance in killing cancer cells with a wide spectrum of cancer cells without the restriction of MHC match.^73^^,^^74^ However, a few studies have suggested they were heavily suppressed in numbers and function in cervical cancer. For instance, a study of 20 patients with stage IA to IIA cervical cancer (cancer group) and 10 women with normal cervix (control group) showed that compared with the control group, NK cells significantly decreased in the cancer group (7.53% ± 4.33% versus 16.00% ± 11.82%, p = 0.035).^75^ Similarly, the analysis of the cervical transformation zone tissue obtained from 19 patients with pathologically confirmed CIN and from 20 patients with normal cervices undergoing hysterectomy for benign indications found that while the distribution of circulating peripheral blood lymphocytes (PBLs) was similar for both patients with neoplasia and normal controls, the percentage of NK cells was significantly low (p = 0.03) in dysplastic tissue as compared to the normal tissue.^64^

Not only numbers, but the NK cell function is also suppressed in cervical cancer by regulatory mechanisms of the patient immune system, especially the Tregs. According to a study, NK cells displayed a significantly higher expression ratio of inhibitory NK receptors (NKRs) (CD158a, CD158b, and NKG2A) and a lower expression ratio of activating NKRs (NKG2D, NKp46, and NKp30) as well as perforin in TILs than PBLs, suggesting suppressed cytotoxicity of NK cells in cervical cancer tumors. The expression ratio of TGF-β1 on Tregs as well as TGF-βRII on Tregs and NK cells was significantly higher in TILs than in PBLs.^76^ Further functional assays demonstrated that NK cell function was suppressed by Tregs, mimicking the inhibition of TGF-β on NK cells, and IL-2/IL-15 stimulation was able to restore NK cell activity.^76^

Additionally, an early study reported the detection of TIA-2, a molecule that reacts with the cytoplasmic domain of the zeta chain in CD3^+^ T and CD16^+^ NK cells. A marked decrease (p < 0.01) in expression of the CD3 ζ chain of PBLs in patients with cervical cancer (n = 22) as compared to PBLs from healthy donors (n = 21) was found. Moreover, PBLs isolated from patients (n = 23) with CIN, to a lesser but significant (p < 0. 01) extent expressed reduced CD3ζ levels as compared to those from healthy donors. This decreased expression of ζ chains was also observed on CD16^+^ NK cells in PBLs from patients with cervical cancer.^77^ These findings suggest that alterations of signal-transducing ζ molecules commonly occur in TILs, including NK cells of patients with cervical cancer, which may relate to their functionality and prognostic value.

### CD4^+^CD25^+^ or FoxP3^+^ Tregs

Both CD4^+^CD25^+^ and FoxP3^+^ Tregs were reported in TILs of cervical cancer. They represent the opposite side of immunity by effectors and negatively regulate effector TIL functions. FoxP3 is the control gene for Tregs, and a study reported the distribution of TILs and FoxP3^+^ cells in cervical cancer (n = 10) and CIN (n = 8) tissues, showing that CD4^+^CD25^+^FoxP3^+^ Tregs accumulated around the tumor cells and that cervical cancer contained a significantly higher proportion of the FoxP3^+^ T cells than found in CIN (p < 0.001).^78^ Moreover, cervical cancer patients with lymph node metastasis had a higher proportion of the FoxP3^+^ T cells than did those without lymph node metastasis (p < 0.05).^78^ This suggests that the increased number of Tregs is important in the immunopathogenesis of cervical cancer and thus has prognostic value in tumor progression. Similarly, another report indicated a significantly higher expression ratio of CD4/CD25 Tregs in TILs than in PBLs of human cervical cancer. Furthermore, *in vitro* co-culture assays illustrated that the expression ratio of TGF-β1 on Tregs as well as TGF-βRII on Tregs was significantly higher in TILs than PBLs,^76^ suggesting that Tregs in TILs are more active.

In addition, a study evaluated the FoxP3^+^ TILs in formalin-fixed paraffin-embedded tissues from 96 cervical cancer patients. The immunostaining density and other clinicopathological features such as FIGO stage, histopathologic type, Ki67 index, HPV status, lymphovasular invasion status, lymph node metastasis, tumor size, stromal invasion status, and parametrial invasion were evaluated for their roles in risk stratification of cervical cancer patients. The patients were stratified into low-, intermediate-, and high-risk groups, and a Spearman’s correlation analysis demonstrated that FoxP3^+^ TILs in the central tumor area showed a statistically negative correlation with risk stratification (p = 0.009).^63^ All of the above data confirmed the prognostic value of Tregs in cervical cancer development.

As to the mechanism of Treg function, a study isolated TILs directly from SCC of the cervix by magnetic-activated cell separation and flow cytometry and sorted them into CD4^+^CD25^+^ Tregs and CD4^+^CD25^−^ (effector T cells [Teffs]) populations. The results showed that 53% of Tregs of cervical cancer were FoxP3^+^ or expressing TGF-β1 and IL-10, and they were able to inhibit the function of both Th (Th1 and Th2) subsets.^79^ Apart from Tregs mentioned in this study, TGF-β1 and IL-10 are also produced by tumor-associated macrophages (TAMs), suggesting that TAMs may also be involved in effector TIL regulation (see Other TILs or infiltrating immune cells).

A recent study evaluated the correlation between Tregs and the viral load of high-risk HPVs in 62 cervical cancer patients assigned into four groups: patients treated with radiotherapy alone or radiotherapy combined with chemotherapy and/or thermotherapy. FoxP3^+^ TILs were detected by IHC, and patients with a high HPV viral load of high-risk HPVs in biopsy tissues showed a significantly lower 15-year survival rate, a higher FIGO stage, and an increased recurrence rate. The distribution of FoxP3^+^ TILs was obviously higher in high HPV viral load groups than in the low viral load groups. A worse clinical outcome was also implicated with increased HPV viral load tested by Cox regression analysis.^80^ Therefore, the presence of Tregs is a negative prognostic indicator for the outcome of patent survival and tumor remission. A metadata analysis in solid tumors indicated that FoxP3^+^ regulatory TILs were not linked to OS of patients with a HR of 1.19 (95% CI: 0.84–1.67),^65^ and the negative prognostic value of Tregs in cervical cancer was demonstrated by Shah et al.,^66^ in which the 5-year survival rate was significantly lower in patients who had a high percentage of FoxP3^+^ Tregs as compared with those who had a lower percentage (35.3% versus 88.9%, p = 0.001).

### Th17 cells

As a minority cell population in TILs, there are not many studies on Th17 cells. However, they may promote tumor progression by fostering angiogenesis and are therefore worth noting. A review article systemically summarized the roles of Th17 cells in cervical cancer and stated that the viral infection alone was not sufficient for the development and progression of premalignant cervical lesions to cancer. The hypothesis was that Th17 cells might be involved in the promotion of uterine cervical cancer (UCC), as high levels of IL-17 expression were detected in the mucosa of the uterine cervix of patients affected by the disease.^81^ Available information suggests that the immune response of Th17 cells during persistent infection of the genital tract with high-risk HPV triggers chronic inflammation with a long duration of the production of IL-17 and other pro-inflammatory cytokines, creating a favorable environment for tumor development. These cytokines are produced by immune cells in addition to tumor cells and appear to function by modulating the host immune system, resulting in an immunosuppressive TME and thus contributing to the growth and progression of the tumor.^81^ Additionally, a study correlated Th17 with Tregs, in which the distribution of Th17 cells was examined in relationship to FoxP3-expressing T cells in TILs from cervical tissues of UCC, CIN, and healthy subjects. Compared with controls, patients with UCC or CIN had a higher proportion of Th17 cells and FoxP3-expressing T cells, and when the ratio of Th17/FoxP3-expressing T cells in TILs was decreased in individual cases, it was more markedly decreased in TILs of patients than in normal cervical tissues. Meanwhile, the cytokine (IL-6, TGF-β, and IL-10) concentrations were significantly higher in UCC patients than those in healthy controls. The levels of intra-tumoral Th17 cells were positively correlated with micro-vessel density in tumors. The authors concluded that the imbalance of Th17/ FoxP3-expressing T cells may play critical roles in the development and progression of UCC and that Th17 cells may promote tumor progression by fostering angiogenesis.^82^ The increased expressions of TGF-β and IL-10 are interesting, as TAMs also produce these cytokines; therefore, whether Th17 cells have any relationship with TAMs is an interesting topic to study. However, different results were reported by Punt et al.,^83^ in which they investigated whether the infiltration levels of Tregs, Th17 cells, and IL-17^+^ cells were correlated with cervical adenocarcinoma patient (n = 67) survival. Intraepithelial, stromal, and total cell frequencies were scored using immunofluorescence methods. The study showed that most Tregs were present in the tumor stroma, while other T cells and IL-17^+^ cells that infiltrated the tumor epithelium were 3-fold more frequent. Low numbers of both Tregs and IL-17^+^ cells were correlated with poor disease-specific survival (p = 0.007). A low number of Tregs combined with Th17 cells present were also correlated with poor survival (p = 0.018). An increased number of IL-17^+^ cells was significantly correlated with the absence of vasoinvasion (p = 0.001), smaller tumor size (p = 0.030), and less infiltration depth (p = 0.021). Their results suggest that Tregs and IL-17^+^ cells represent a beneficial immune response, whereas Th17 cells might represent a poor response in cervical adenocarcinoma. Because of the results, more detailed studies on Th17 and IL-17 functions in cervical cancer development are needed.

### γδ T cells

A special subset of T cells is γδ T cells (Figure 2). Their number and presence indicate a positive prognostic sign for patents. A study investigated the distributions of lymphocyte subsets in tumor tissues and peripheral blood samples from cervical cancer patients and patients with precancerous lesions. A total of 44 patients with stage IB1 to IIA2 cervical cancer and 13 patients with precancerous lesions were included, and the total amounts of T lymphocytes and granulocytes were significantly higher in the tumor tissue than in the peripheral blood in the cervical cancer patients, while γδ T cells exhibited the opposite trend (p < 0.05),^84^ suggesting that a decreased γδ T cell number is associated with cancer progression. In addition, γδ T cells have a specific ability to kill HPV^+^ cells as demonstrated by Li et al.,^85^ where they analyzed the antitumor activity of *ex vivo*-expanded γδ T cells derived from TILs of cervical cancer patients using both *in vitro* and *in vivo* models. The percentage of T cell receptor (TCR) γδ^+^ cells in γδ TILs after *ex vivo* expansion was 91.2% ± 1.2%, and they demonstrated marked cytotoxicity to SiHa or HeLa cells. Moreover, the cytotoxicity of γδ TILs toward SiHa or HeLa cells was significantly increased when effector and target cells were incubated with either lactose or galectin-1 antibody at an effector-to-target (E:T) ratio of 1:1 (p < 0.05), as SiHa cells expressed and secreted galectin-1. In addition, γδ TILs, in combination with galectin-1 antibody treatment, significantly suppressed the growth of tumor xenografts in severe combined immunodeficiency (SCID) mice (p < 0.05), although γδ TILs alone showed the ability to inhibit tumor growth *in vivo*.^85^ Thus, the *in vitro* expansion of this specific cell population may be necessary for their clinical application as TIL therapy.

### Other TILs or infiltrating immune cells

#### B cells

B cells were reported to be present in TILs by Sheu et al.^75^ This study quantitatively measured and compared T and B cell infiltration into the normal (10 women with normal cervix, control group) and neoplastic cervix (20 patients with stage IA to IIA cervical cancer, cancer group). The isolated mononuclear cells from tissue specimens were stained and analyzed by flow cytometry. Compared with the control group, lymphocytes isolated from cancer tissue consisted of higher proportions of B cells (CD19^+^) (7.23% ± 4.49% versus 3.67% ± 3.19%, p = 0.016) and T cells (72.33% ± 8.70% versus 53.15% ± 17.36%, p = 0.004). However, the functional and prognostic roles of these B cells were not described and are the subject of further investigations.

#### Macrophages

The TME plays essential roles in cancer development, and TAMs are a major player in the TME.^86^^,^^87^ Their presence therefore has prognostic values, especially as their inhibitory cytokines, such as IL-10 and TGF-β, influence T cell (TIL) functions.^86^^,^^88^ A study investigated the density of TAMs in intraepithelial and tumor stromal areas of 148 patients with cervical adenocarcinoma, who were divided into two groups showing high and low cell infiltration, using the median value as a cutoff. Most cases (54.7%) were classified in stage IBb and 26 (17.6%) had recurrent disease. The density of stromal CD68^+^ or CD204^+^ macrophages that had infiltrated invasive adenocarcinoma (n = 127) was significantly higher than in adenocarcinoma *in situ* (n = 27). The Kaplan-Meier survival analyses revealed that a higher density of tumor-infiltrating CD204^+^ M2 macrophages was significantly associated with shorter disease-free survival (p = 0.0027).^89^ However, another study reported different types of CD68^+^ and CD163^+^ TAMs in formalin-fixed paraffin-embedded tissues from 96 cervical cancer patients and showed that no significant correlation was found between the presence of the TAMs with FIGO stage, LVI status, status of lymph node metastasis, parametrial invasion, stromal invasion status, and tumor size.^63^ It seems that not all types of TAMs have prognostic values, but tumor-infiltrating CD204^+^ M2 macrophages are a prognostic factor for patients with cervical adenocarcinoma. As mentioned above (see NK cells), TAM function has a similarity to Tregs, so both can be considered together in TILs. However, note that in therapeutic TILs, macrophages are not included.

### TIL ratios with prognostic values

TILs consist of different cell populations, and their composition, especially the ratio between subsets, has prognostic values and reflects the immune function dominance and variations during cervical cancer development.

#### CD4/CD8 ratio

The CD4/CD8 ratio is the most reported in TILs, and the available data are consistent with the CD4^+^ number analysis described above. For example, a study analyzed the infiltration of CD4^+^ and CD8^+^ lymphocytes in cervical cancer tissue and peripheral blood of Indian women during a period of 12 months. They found that compared to peripheral blood, CD4^+^ cells were significantly less predominant in tumor tissue (p = 0.0013) and there was a statistically significant (p = 0.0004) reversal of the ratio of CD4^+^ and CD8^+^ cells in the tumor tissue (0.68 ± 0.39) compared to peripheral blood (1.5 ± 0.66), with maximal alteration in higher stage disease.^90^ Moreover, the CD4/CD8 ratio was significantly lower in tumors from patients with lymph node metastasis (n = 8) than in those without lymph node metastasis (n = 22, 0.50 versus 0.81, p = 0.001) and was much lower in bulky tumors (tumor size >4 cm, n = 5) than in non-bulky tumors (n = 25, 0.41 versus 0.81, p = 0.001).^67^ In addition, a higher CD4/CD8 ratio was significantly associated with the higher patient 5-year survival rate (82.4% versus 44.4%, p = 0.029) and was significantly lower in patients who died as compared with those who survived (0.60 ± 0.25 versus 1.17 ± 1.02, p = 0.019).^66^ All of these data suggest that the reversed CD4/CD8 ratios are correlated with rapid tumor growth, lymph node metastasis, and finally the poor clinical outcome of patients with cervical carcinoma.

#### CD8^+^/Treg ratio

In contrast to CD4/CD8, this ratio represents a positive prognostic sign for better patient outcomes. Alterations of TILs before and after NACT and their prognostic significance in advanced cervical cancer patients treated with platinum-based NACT were reported with 137 patients at stage IB2 and IIA2 cervical cancer. Pre-treatment biopsy and surgical specimens after NACT were examined for CD8 and FoxP3 TILs. The CD8^+^ T cell counts remained stable in both intra-tumoral (median, 121.32 versus 109.59; p = 0.414) and peritumoral (median, 402.56 versus 390.84; p = 0.255) areas, but multivariate analyses demonstrated that the high ratio of intra-tumoral CD8/peritumoral FoxP3 in residual tumors was an independent prognostic factor for both PFS (HR = 0.297; 95% CI: 0.109–0.810; p = 0.018) and OS (HR = 0.078; 95% CI: 0.010–0.598; p = 0.014)^91^. This study suggests that a decrease of FoxP3 TILs or an increase of intra-tumoral CD8/peritumoral FoxP3 ratio after NACT may confer a favorable clinical outcome. In agreement with the assumption, a study investigated the effect of intraepithelial TILs (ieTILs) and their ligands expressed by cervical tumor cells on the outcome of 115 cervical cancer patients. T cell subsets, CD57^+^ cells, and Tregs were examined. The associations of these different ieTIL subtypes with HLA class I and MHC class I chain-related molecule A (MICA) expression were determined in relationship to clinical variables and patient survival. Survival analysis showed that a high number of intraepithelial Tregs (FoxP3^+^), a low CD8^+^/Treg ratio, and a weak HLA-A expression were all associated with worse survival (p = 0.034, 0.025, and 0.033, respectively). Further stratification of patient groups based on HLA-A-MICA expression and the HLA-A-MICA-CD8^+^/Treg ratio revealed an even poorer survival (p = 0.005). In a multivariate Cox analysis, a low CD8^+^/Treg ratio (p = 0.047), weak HLA-A-MICA expression (p = 0.003), and weak HLA-A-MICA expression combined with a low CD8^+^/Treg ratio (p = 0.002) were all found to be independent unfavorable predictors in cervical carcinoma (HR = 2.7, 4.0, and 4.9, respectively).^92^

## Therapeutic TILs of cervical cancer

### General procedure of TIL expansion

**Figure 3:**
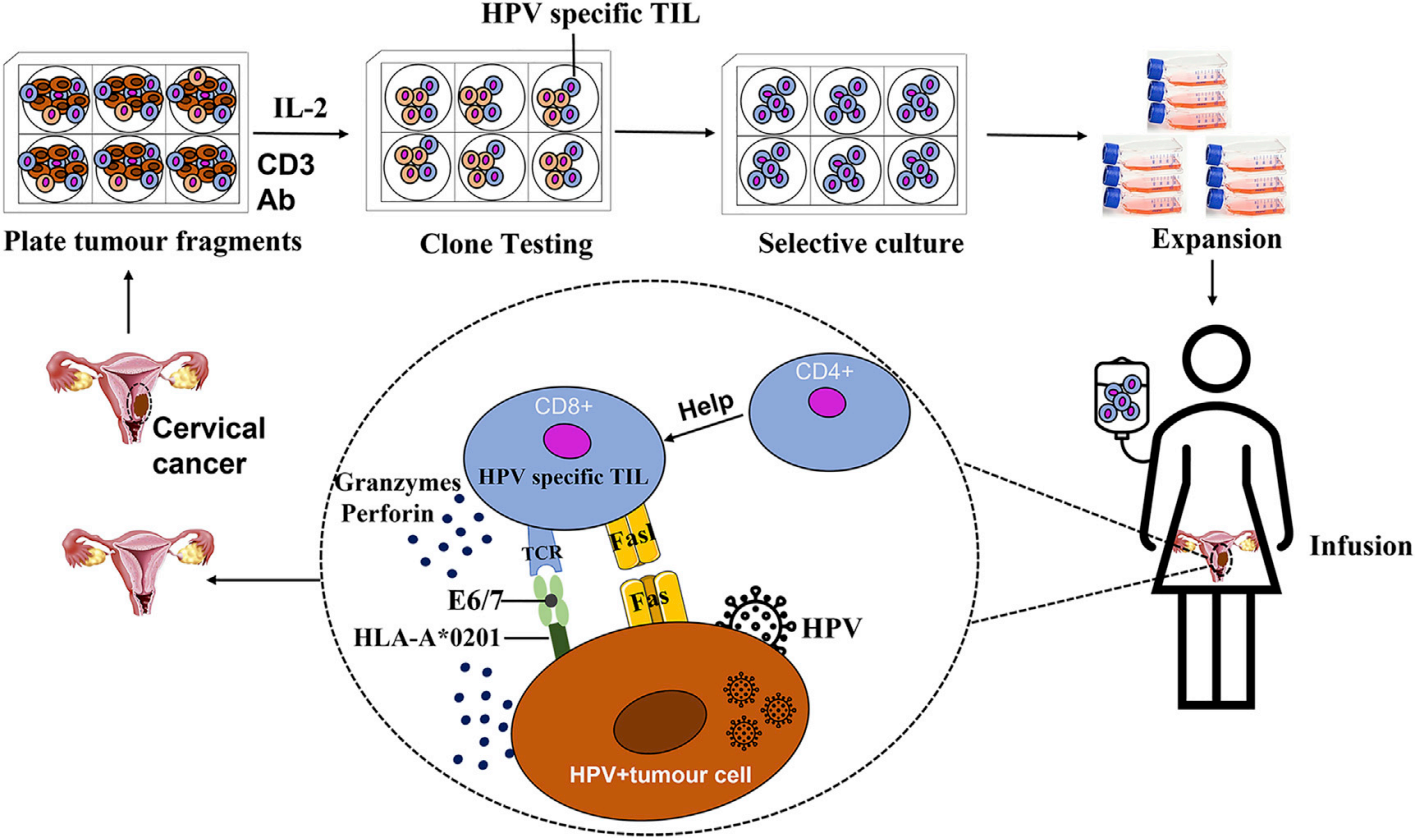
The symbolic procedure of TIL therapy in cervical cancer Tumor tissues are mechanically and enzymatically isolated into single cells and seeded into plates in the presence of IL-2 and CD3 antibody (CD3-Ab) for proliferation into TILs. TILs may be further selected for reactivity to HPV E6/E7 and *in vitro* expansion before reaching enough numbers for patient infusion. HPV-specific TILs will utilize their unique mechanisms to attack or kill tumor cells (HPV-tumor cell).

The *in vitro* expansion of TILs was first proposed by researchers at the NIH.^32^^,^^93^ The production process was divided into several typical steps: the tissue adjacent to the tumor was collected during surgery or biopsy with a diameter of about 0.5–1 cm; after rinsing with 0.9% physiological saline, the tumor sample was transported to a proprietary GMP facility; tumor tissues were mechanically diced and placed in cell cultural plates; and TILs were separated and propagated with a high concentration of IL-2 for selective cultivation. During the cultivation, TILs could be further screened for specific populations for selective expansion to required numbers. At the same time, the patients began a week of pretreatment (myeloablative) to prepare for TIL infusion (Figure 3). Immediately after the TIL infusion, up to 6 doses of IL-2 were received to support the growth and activation of the transferred TILs. Similar to other cancers, advances have been made in TIL immunotherapy for cervical cancer. According to the study type, they are classified into three categories, including clinical trials, *in vitro* studies, and animal studies.

### Clinical trials

Only a few clinical studies have been documented, and a milestone study by Stevanović et al.^94^ reported TIL therapy in patients diagnosed with metastatic cervical cancer who had previously received platinum-based chemotherapy or chemoradiotherapy. The patients were treated with a single infusion of TILs (around 1 × 10^10^ cells) selected whenever possible for HPV E6 and E7 reactivity (HPV-TILs, using an enzyme-linked immunospot [ELISPOT] assay). Cell infusion was preceded by lymphocyte-depleting chemotherapy and followed by administration of aldesleukin (or IL-2). The results showed that three of nine patients experienced objective tumor responses (two complete and one partial). The two complete responses were ongoing 22 and 15 months after treatment, respectively. One partial response was 3 months in duration. The HPV reactivity of T cells in the infusion product (measured by IFN-γ production and CD137 upregulation) correlated positively with the clinical response (p *<* 0.0238). In addition, the frequency of HPV-reactive T cells in peripheral blood 1 month after treatment was also positively associated with the clinical response (p < 0.0238). As reported by the same group, TIL therapy of patients with metastatic HPV-associated carcinomas was evaluated. The trial was a phase II design with two cohorts, that is, cervical cancers and noncervical cancers. Cell infusion was preceded by lymphocyte depletion, followed by a systemic high dose of aldesleukin. Objective responses occurred in 5 of 18 (28%) patients in the cervical cancer cohort and 2 of 11 (18%) patients in the noncervical cancer cohort. Two of the responses in cervical cancer were complete and were ongoing 67 and 53 months after treatment. Responses in the noncervical cancer cohort were in anal cancer and oropharyngeal cancer. The HPV reactivity of the infused T cells correlated with the clinical response. Peripheral blood repopulation with HPV-reactive T cells also correlated with the clinical response.^95^ Although the cohort sizes of the two above studies were small, the results are promising and confirm that TIL therapy can provide a new treatment approach for cervical cancer.

### *In vitro* studies of HPV-specific TILs

Apart from the positive role of HPV-reactive TILs in clinical trials, their specificity to HPV was further investigated in *in vitro* studies. Using HPV E6–E7 proteins/peptides, including their expressions of MHC alleles, Oerke et al.^96^ reported the overrepresentation of the HLA-B8 allele (28.44%) in cervical cancer patients as compared to the MHC class I allele frequency in a healthy control population (18.80%) and the identification of an HLA-B8-binding peptide TLHEYMLDL HPV16 E7(7–15), which can drive HPV16 E7-specific and MHC class I-restricted T cell responses in PBLs from healthy individuals. In addition, TLHEYMLDL-specific T cells recognized the naturally processed and presented peptide on HPV16^+^ cervical cancer cells transfected with the HLA-B8 gene defined by IFN-γ production. Moreover, this peptide epitope was also recognized by freshly harvested TILs or T cells from tumor-draining lymph nodes (T-LNs) from patients with cervical cancer determined by both flow cytometry and tetramer *in situ* staining,^96^ suggesting that this peptide epitope is a useful sequence to identify therapeutic TILs in cervical cancer.

A study has defined the nature of the cellular immune response in five women with HPV16^+^ cervical carcinoma using two approaches of tetramer-guided technology, including (1) the *in situ* localization of MHC class I peptide complexes in the tumor lesions, and (2) the *ex vivo* sorting of HLA-A∗0201-restricted and HPV16 E6-reactive T cells. CD8^+^ T cells from the PBLs, TILs, and T-LNs were analyzed. HPV16 E6 tetramer-sorted lymphocytes from the different anatomic sites recognized an HLA-A∗0201-restricted E6 peptide regardless of the type of antigen-presenting cells used for stimulation as determined by IFN-γ production: by autologous tumor cells, by HLA-A∗0201 surrogate antigen-presenting cells pulsed with the small peptide, and by an HLA-A∗0201-matched human DC line transgenic for HPV16 E6. Further analysis showed that the HPV16 E6-reactive CD8^+^ T cells were of high avidity defined by blocking with an anti-CD8α-specific monoclonal antibody.^97^

Furthermore, a study reported the analysis of the presence and specificity of cervix-infiltrating and T-LN-resident T cells in a group of 74 patients with cervical malignancies. Among the 54 HPV16^+^ or HPV18^+^ patients, the presence of HPV16- or HPV18-specific T cells was detected in at least 23 patients but not in the 20 controls. The study also identified 17 novel CD4^+^ and CD8^+^ T cell epitopes and their HLA restriction peptides and revealed that the HPV-specific immune response was against both E6 and E7 and showed no preferential recognition of immunodominant regions. Unexpectedly, the vast majority of the CD4^+^ T cell epitopes were presented in the context of the less abundantly expressed HLA-DQ and HLA-DP molecules.^98^ Since the identified T cell epitopes constitute physiological targets in the immune response to HPV16^+^ and HPV18^+^ tumors, they will be valuable for detailed studies on the interactions between the tumor and therapeutic TILs.

To further prove the critical role of MHC class I matching, a study reported culturing and cloning TILs from a patient with cervical carcinoma and examined the *in vitro* characteristics of these TILs with an established autologous tumor cell line. T cell clones were generated in the presence of 20 U/mL recombinant IL-2 and irradiated autologous tumor cells, PBLs, and EBV-transformed B cell lines. All clones were CD3^+^/CD8^+^ and cytolytic against autologous tumor cells, but did not lyse autologous lymphoblasts, with a minor lytic capacity on allogeneic cervical tumor cell lines or tumor cell lines of other histologic types. Cytotoxicity against the autologous tumor could be inhibited by anti-CD3, anti-CD8, and anti-ICAM1 but not by anti-HLA class I (W6/32, B9.12.1), anti-allele-specific HLA determinants, and anti-LFA-3 antibodies.^99^ This study suggests a highly specific autologous lytic activity of cervical carcinoma TILs, in which a CD3-associated surface antigen recognition is involved.

As to whether there is a cross-reactivity of TILs between the major HPV types 16 and 18, a study tested the HPV type of the tumors from 65 consecutive patients with cervical cancer and immunologically assayed HPV cross-reactivity on all available archived samples of oncoprotein-reactive TILs from HPV^+^ tumors (n = 16) and on a library of previously identified TCRs (n = 10). The HPV genotype of each patient’s tumor was determined and the cross-reactivity of archived TILs and a library of TCRs was assessed. The results showed that 39 of 65 patients (60.0%) had HPV16^+^ tumors and 21 patients (32.3%) had HPV18^+^ tumors. In the analysis of cross-reactivity, 1 of 16 oncoprotein-reactive archived TILs (9 from cervical cancers and 7 from other cancers) displayed HPV16/HPV18 cross-reactivity. None of the 10 oncoprotein-reactive TCRs displayed HPV16/HPV18 cross-reactivity. The results suggested that HPV16/HPV18 intergenotype T cell cross-reactivity of T cells from HPV16^+^ and HPV18^+^ cancers was uncommon.^100^ Summarizing the *in vitro* studies above, it seems that MHC class I matching is important for therapeutic TILs of cervical cancer. Additionally, A∗020-1 and others in the B8 allele may also be important, and these TILs are HPV type specific (Figure 3).

Apart from tumor tissues, TILs can be isolated from the T-LNs as reported by van Poelgeest et al.^101^ In their study, lymphocytes of 11 patients with cervical cancer were expanded in the presence of HPV16 E6 and E7 synthetic long peptides and IL-2. On average, a 36-fold expansion of a CD4^+^ and/or CD8^+^ HPV16-specific T cell population was achieved, and they maintained the capacity for secondary expansion. The Th1 cytokine IFN-γ was produced in all cell cultures, and in some cases also the Th2 cytokines such as IL-10 and IL-5.

### Animal models

Some promising results were obtained from *in vivo* animal model studies. Li et al.^85^ reported that γδ TILs, in combination with galectin-1 antibody, significantly suppressed the growth of xenograft cervical tumor in SCID mice, in comparison with control groups (p < 0.05), although γδ TILs alone already showed the ability to inhibit tumor growth. In addition, TCR-targeted therapy is a promising cancer treatment and may depend on the identification of high-avidity TCRs against tumor-restricted target antigens. Therefore, HPV16 E7-specific, HLA-A∗02:01-restricted TCRs from a uterine cervix biopsy of a woman with CIN was demonstrated to have high functional avidity and CD8 coreceptor-independent tumor targeting. Human T cells transduced to express the TCR specifically recognized and killed HPV16 ^+^ cervical (CasKi cells) and oropharyngeal (SCC 90 and SCC 152, two head and neck cancer cell lines) cancer cell lines and mediated regression of established HPV16^+^ human cervical cancer tumors in a mouse model.^102^

A study reported an interesting investigation of the SCID mouse model, including HPV16^+^ SiHa subcutaneously transplanted (SiHa-SCID), human PBL intraperitoneally transplanted (Hu-PBL-SCID), Hu-PBL intraperitoneally and SiHa subcutaneously transplanted (Hu-PBL-SiHa-SCID), and PBS subcutaneously transplanted (PBS-SCID) mice as the control. The transplanted tumor grew slowly, and no metastasis was found in Hu-PBL-SiHa-SCID mice. The survival time of Hu-PBL-SiHa-SCID mice was significantly longer than that of SiHa-SCID mice. The numbers of human CD3^+^, CD4^+^, and CD8^+^ T cells were significantly increased in the peripheral blood and spleen of Hu-PBL-SiHa-SCID and Hu-PBL-SCID mice, and significantly higher in Hu-PBL-SiHa-SCID mice than those of Hu-PBL-SCID mice. TILs of human CD4^+^ T cells were detected in Hu-PBL-SiHa-SCID but not in SiHa-SCID mice. The spleen cells of Hu-PBL-SiHa-SCID mice displayed significantly stronger cytotoxicity to target cells than those of SiHa-SCID mice.^103^ Although CD8^+^ TILs were not directly detected, the study suggests that a HPV16^+^ cervical carcinoma model can be established in SCID mice and a spontaneous anti-tumor immune response to human cervical cancer can be present in transplant tumor after the human immune system is reconstructed.

### Efficacy of TIL therapy in other cancers

#### Clinical studies

Apart from cervical cancer, the efficacy of TIL therapy has been well established in other epithelial cancers. As mentioned above, TIL treatment showed promising results of tumor regression in patients with metastatic melanoma after administration of TILs with IL-2.^15^ Rosenberg’s team also reported the TIL treatment of 88 patients with stage III melanoma and showed that the relapse-free survival period of patients receiving TIL was significantly prolonged.^104^ In 2016, Rosenberg and colleagues^34^ evaluated the intensity of lymphocyte clearance in patients with metastatic melanoma before TIL treatment. The results showed that TILs can mediate tumor regression in most patients with metastatic melanoma (52/93; 56%). Apart from studies from Rosenberg and colleagues, several other studies demonstrate the efficacy (lasting effect or complete remission) of TIL therapy in treating refectory melanoma,^105^^,^^106^ advanced melanoma,^107^ and metastatic melanoma.^108^, ^109^, ^110^

In other cancers, 29% of kidney cancer patients achieved objective tumor responses with the treatment of autologous TILs and continuous IL-2 infusion.^111^ Aoki et al.^112^ reported a TIL treatment trial for patients with advanced or recurrent ovarian cancer, in which 5 of 7 had clinical efficacy, and among TIL-treated patients who received chemotherapy, 9 of 10 had clinical efficacy. However, in contrast, Freedman et al.^113^ reported the injection of IL-2 to 11 patients with ovarian cancer after intraperitoneal injection of TILs showed no clinical response. The difference between the two studies is not clear but may be related to the differences in treatment regime, TIL injection route, TIL colleting time and expansion conditions, and other factors . In 2015, Jiang et al.^114^ treated 15 patients of primary liver cancer with activated and expanded TILs after tumor resection. They stated that activated and expanded autologous TIL immunotherapy is a low-toxic, effective new method for the treatment of HCC. TIL infusion can be combined with other anti-cancer methods to enhance the cancer killing effect and provide treatment for refractory tumors. At the 2020 American Association for Cancer Research (AACR) meeting, the Moffett Cancer Research Center of the United States announced the phase I clinical trial results of TIL therapy carried out in 12 patients with advanced non-small cell lung cancer that had undergone PD-1 (Opdivo [nivolumab]) treatment. It was reported that TIL therapy can achieve a total response rate of 25%, and two patients achieved lasting complete remission. Despite the small size of the study, oncologists are still excited because TIL provides a treatment path for the most refractory patient group, and TIL therapy prolongs the survival of patients with PD-1-resistant non-small cell lung cancer.

In 2009, Besser et al.^115^ treated refractory metastatic melanoma with young TILs, and the results showed tumor regression in more than half of the patients with refractory metastatic melanoma and no deaths related to the treatment. Using the same unscreened CD8^+^ enriched young TILs, Rosenberg and colleagues^32^ found that a fast and simple method can reliably prepare CD8^+^-enriched young TILs as a method for the treatment of advanced melanoma with better outcomes. The analysis of the 62-month follow-up of 93 patients with metastatic melanoma found that autologous young TIL cell transplantation for the treatment of metastatic melanoma can mediate a lasting complete response, and its efficacy is similar regardless of the previous treatment (chemotherapy or radiotherapy).^33^ Itzhaki et al.^116^ optimized the young TIL production process and confirmed a significant correlation between short culture time, high cell number infused, and tumor regression. In summary, TILs have shown good efficacy in a variety of refractory tumors. Unlike CAR-T and NK cell therapy for tumors, TILs have a stronger ability to kill tumor cells. Immune cells of TILs are derived from tumor tissues, while most other cellular immunotherapies come from the blood, which directly determines the ability of immune cells to recognize tumors.

#### Preclinical and *in vitro* studies

In 1986, Rosenberg et al.^16^ reported a C57BL/6 mouse metastasis model, where 4 × 10^6^ to 5 × 10^6^ TILs combined with IL-2 could eliminate 96% of micro-metastases, and under the combined action of cyclophosphamide and TILs, together with IL-2, had a cure rate of 100% for late liver metastasis and 50% for lung metastasis in mice (n = 12) carrying MC-38 colon adenocarcinoma. Topalian et al.^117^^,^^118^ also reported the isolation of TILs from patients with melanomas, sarcomas, and adenocarcinoma. After expansion and culture, they found tumor-specific CTL subgroups in non-specific TIL cultures and the *in vitro* experiment in autologous tumor cells confirmed their effective killing ability. These studies provide evidence for cancer patients with MHC limited specific immune responses to autologous tumors and have significance for clinical trials of TIL immunotherapy for various malignancies.

In 2008, Rosenberg and colleagues^119^ established a young TILs method, and analysis of lymphocyte subsets in TILs showed that the frequency of CD4^+^ lymphocytes in young TILs was higher than that of standard TILs. However, there was no significant difference in the frequency of CD8^+^ cells between young and standard TILs. The frequency of CD3^−^CD56^+^ NK cells in young TILs was low. Goff et al.^120^ processed 787 tumors from 402 patients to generate TILs, and 376 patients (94%) developed TILs, in which active-specific TILs were detected in 269 patients (67%). The study also found that sex and age were not related to TIL growth and activity. HLA-A2 status had nothing to do with TIL growth, but A201^+^ tumors were more likely to show TIL activity. In addition, it is more difficult to produce active TILs in gastrointestinal metastases, while tumors from lung and lymph node metastases are more likely to produce active TILs. In a different manner, Radvanyi and colleagues^121^ found that parameters such as age, sex, type and timing of systemic treatment significantly affected the growth of TILs. Ben-Avi et al.^122^ compared TILs derived from lung tissue with TILs derived from other sources and found that the success rate of establishing TIL culture from lung tissue is significantly higher than that of non-lung tissue. Moreover, Nielsen et al.^123^ showed that by adding agonist 4-1BB and OKT3 antibody can greatly increase the production of TILs and halve the culture time required to produce enough TILs.^124^ The variations of the above-mentioned studies reflect that it may be necessary to develop a unique protocol for each patient with different tissue.

### Toxicity of TIL therapy

Generally, the toxicity or side effect of TIL therapy is light or moderate and majorly associated with a lymphodepleting regimen and IL-2 administration. There is no such report on cervical cancer, although there are a few on other cancers. Dudley et al.^125^ first described the toxicity of TIL therapy of 35 patients with metastatic melanoma. The patients all underwent lymphodepletion with 2 days of cyclophosphamide (60 mg/kg) followed by 5 days of fludarabine (25 mg/m^2^). They then received cell infusion with autologous, rapidly expanded TIL cultures and high-dose IL-2. Toxicities observed included the expected hematologic toxicities of chemotherapy such as neutropenia, thrombocytopenia, and lymphopenia, as well as the transient toxicities of high-dose IL-2 therapy, with two patients who developed pneumocystis pneumonia and one patient who developed Epstein-Barr virus-related lymphoproliferation. Similarly, Yeh et al.^126^ reported that TILs plus IL-2 therapy after a lymphodepleting regimen of cyclophosphamide and fludarabine induced early high fever, diffuse rash, hearing loss, and bilateral anterior uveitis, which developed acutely in the patients with metastatic melanoma, and late autoimmune sequelae included the development of alopecia, vitiligo, poliosis, and bilateral panuveitis with diffuse retinal pigment epithelium (RPE) hypopigmentation, reminiscent of Vogt-Koyanagi-Harada (VKH) syndrome. Chandran et al.^127^ further reported toxicities of TIL therapy in patients with metastatic uveal melanoma. They found that grade 3 or worse toxic effects were related to the lymphodepleting chemotherapy regimen, including lymphopenia, neutropenia, and thrombocytopenia (21/21); anemia (14/21); and infection (6/21).

In contrast to melanoma cases, in hepatocellular carcinoma (HCC), TIL therapy seemed to not cause too much toxicity in the patients. The study reported by Jiang et al.^114^ showed that 15 HCC patients were treated with activated and expanded TILs following tumor resection. A transient increase in the frequency of T cells was observed after adoptive transfer and was found to be associated with grade I flu-like symptoms and malaise. After a median follow-up of 14 months, 15 patients (100%) were alive, 12 patients (80%) showed no evidence of disease, and 3 patients had tumor recurrence. Similarly, in lung cancer, adverse effects of TIL therapy were reported to be mild, as only 3 of 15 patients had fever, headache, and nausea immediately after infusion and then recovered within several hours, whereas other patients had no side effects. Moreover, the immunity function of all patients was improved after infusion.^129^ It seems that the toxicity of TIL therapy may vary with the cancer type. However, because TIL therapy is usually accompanied with high-dose IL-2 administration, the cause of some general side effects can be attributed to IL-2. Systemic IL-2 administration has had a long history of high regression rates in patients with various advanced cancers.^130^^,^^131^ The high-dose IL-2 itself could exert a variety of serious dose-dependent systemic toxicities. One of the most predominant toxicities is the capillary leak syndrome, caused by extravasation of fluid into the organs, causing multi-organ damage.^132^ These biologic effects are the result of IL-2 activation of NK and other immune cells, which in turn stimulate the release of inflammatory cytokines and eventually lead to the so-called cytokine storm.^133^ The cytokine storm is a major driver of the IL-2-mediated side effects, some of which include fever, chills, malaise, diarrhea, nausea, anemia, thrombocytopenia, eosinophilia, elevation of hepatic enzymes, and confusion.^134^ An increase in the vascular permeability may be caused by IL-2 and CD25-dependent endothelial cell damage and indirectly by the release of NK cell-mediated tumor necrosis factor (TNF)-α.^135^^,^^136^ High-dose IL-2 has also been linked to the development of eosinophilic myocarditis.^137^ It can also enhance neutrophil chemotaxis, which makes patients who receive IL-2 more prone to Gram-positive and Gram-negative organism infections.^138^ Due to this complexity of IL-2, appropriate care and management of treated patients is crucial. For example, it was suggested that patients should be initially screened for any cardiac pathology, and those found to have a cardiac abnormality should not be favored for IL-2 therapy.^139^ Apart from IL-2, a review article summarized four types of general toxicity of ACT that are also relevant to TIL therapy to some extent.^140^

## Methods to enhance TIL therapy

### Selective expansion of specific and neoantigen TILs

As mentioned above, TILs contain T cells reactive to cervical cancer cells or HPV^+^ cells.^94^ These cells or clones can be selectively expanded for enriched TIL therapy. For example, the special selection of HPV E6/E7-specific^94^ and γδ TILs^85^ can increase the efficacy of TIL therapy. In addition, there is a very important population of TILs that recognizes tumor neoantigens that should be considered for selective expansion. Neoantigens are derived from gene mutation and gene fragment insertion or deletion of tumor cells as the major and specific tumor antigens,^141^ and they have attracted increasing interests for their potential in cancer immunotherapy. Since TILs closely contact tumor cells, they are assumed to react quickly and sensitively to neoantigens. Indeed, emerging evidence suggests that neoantigen-specific TILs are the major effector cells that mediate tumor regression after checkpoint inhibition in different cancers.^142^, ^143^, ^144^ Preliminary analysis has also indicated that the number of neoepitopes identified in melanoma patients was associated with the number of nonsynonymous somatic mutations identified in patient tumors but was not associated with response to therapy,^18^ suggesting that the somatic mutation is the major source of neoantigens. Neoantigens have been identified in several types of cancers.^18^^,^^141^ For cervical and other viral-induced cancers, viral antigens are considered as neoantigens.^141^ Regardless of whether the viral antigens are tumor-specific antigens or neoantigens, targeting them is a better strategy, as they are foreign and selective expansion of TILs reactive to viral antigens or variants will increase the efficacy of TIL therapy with increased safety. A fundamental challenge for the neoantigen approach is to effectively identify and isolate neoantigen-specific T cells or their TCRs, although several new strategies have been proposed.^145^ A new approach included the co-culture of TILs with tandem minigene (TMG)-transfected or peptide-pulsed autologous APCs, and the single-cell RNA sequencing analysis of T cells to identify paired TCR sequences associated with cells expressing high levels of IFN-γ and IL-2 was reported.^146^ Using high-throughput immunologic screening of mutant gene products identified via whole-exome sequencing was also reported for common gastrointestinal cancers.^19^ Use of the mass spectra method to identify neoepitopes binding to MHC molecules of reactive TILs is proposed as a more practical and quick way to identify neoantigens.^18^ However, due to the heterogeneous nature of tumor cells, neoantigen-enriched TILs reactive to a limited number of neoepitopes may not be sufficient to eliminate whole tumor growth,^141^ suggesting that more understanding of the neoantigen development and immune responses against them are necessary.

### DC vaccine further boost

A study evaluated the potential of autologous DCs pulsed with HPV16 and HPV18 E7 oncoprotein in restoring tumor-specific cytotoxicity in populations of TILs for adoptive immunotherapy of cervical cancer patients. Full-length E7-pulsed DC-stimulated CD8^+^ T cells derived from PBLs and from tumor tissues (TILs) were tested and compared for their ability to induce an HLA class I-restricted CTL response against autologous tumor cells. DC stimulation induced powerful cytotoxicity against autologous tumor target cells by TIL-derived CD8^+^ T cells from all three cervical cancer patients, while autologous Epstein-Barr virus-transformed lymphoblastoid cell lines were not lysed. Killing of autologous tumor cells was higher by CD8^+^ T cells from TILs compared to PBLs (p > 0.01) and was more strongly inhibited by anti-HLA class I monoclonal antibody (mAb) (p > 0.05). Phenotypically, all CTL populations were CD3^+^/CD8^+^, with higher levels of CD56 expression by TIL-derived CTLs and TIL-derived CD8^+^ T cells having shown a higher percentage of IFN-γ^+^ cells compared to PBLs.^147^ This study suggests that DC-stimulated TILs may represent a superior source of tumor-specific CTLs for TIL therapy of patients harboring metastatic or recurrent cervical cancer refractory to standard treatments. Alternatively, a study reported the use of a viral gene delivery platform carrying HPV16 genes E6 and E7 (Ad5 [E1-, E2b-]-E6/E7) to boost the therapeutic effect, particularly CD8^+^ TILs in cervical cancer. Immunotherapy using Ad5 [E1-, E2b-]-E6/E7 resulted in clearance of small tumors and an OS benefit in mice with larger established tumors. When immunotherapy was combined with immune checkpoint PD-1 blockade, an increased level of anti-tumor activity against large tumors was observed. Analysis of the TME in Ad5 [E1-, E2b-]-E6/E7-treated mice revealed elevated CD8^+^ TILs.^148^ This suggests that *in vivo* boosting of TILs is also possible.

### Immune checkpoint inhibition

Immune checkpoints are molecules expressed on immune cells such as T or B cells and APCs to negatively regulate immune responses.^6^^,^^7^ The presence of these molecules, especially with high-level expression, is an indicator of immune function suppression. For cervical cancer, a review article systemically analyzed 126 published papers and concluded that the expression of PD-1/PD-L1 was associated with HPV-related cancers, especially with HPV16^+^ and HPV18^+^ cases. The reason was that HPV E5/E6/E7 oncogenes activated multiple signaling pathways, including phosphatidylinositol 3-kinase (PI3K)/AKT, mitogen-activated protein kinase (MAPK), HIF1α, STAT3/nuclear factor κB (NF-κB), and microRNAs, which regulated the PD-1/PD-L1 axis to promote HPV-induced cervical carcinogenesis. The PD-1/PD-L1 axis then played a crucial role in immune escape of cervical cancer through inhibition of host immune response creating an “immune-privileged” site for initial viral infection and subsequent adaptive immune resistance.^3^ This analysis provides a rationale for therapeutic blockade of the PD-1/PD-L1 axis for HPV^+^ cancers. Indeed, these molecules can be inhibited for enhancing immune or TIL functions via either blockade antibodies or gene silencing, which are widely published in clinical practice.^6^^,^^149^^,^^150^ A study by us also demonstrated that gene silencing either PD-1 or PD-L1 or both can significantly increase the cytotoxicity of TILs from breast cancer patents.^151^ We think this is also true in cervical cancer, which is under investigation.

#### Immune checkpoint expression in TILs of cervical cancer

A study comprehensively analyzed eight immune checkpoints on TILs from 172 primary cancer patients (131 were blood-tumor-matched patients) covering the eight most prevalent types of cancers, including cervical cancer. The eight immune checkpoints were PD-1, cytotoxic T lymphocyte antigen-4 (CTLA-4), T cell immunoglobulin and mucin-domain containing-3 (Tim-3), 2B4, killer cell lectin like receptor G1 (KLRG-1), TIGIT, B and T lymphocyte attenuator (BTLA), and CD160. The result showed an increased expression of PD-1 and Tim-3 but a decreased expression of BTLA on TILs when compared with peripheral blood from multiple types of cancer. The co-expression analysis of key immune checkpoint receptors delineates “shared” subsets as PD-1^+^Tim-3^+^TIGIT^+^2B4^+^KLRG-1^−^CTLA-4^−^ and PD-1^+^TIGIT^+^2B4^+^Tim-3^−^KLRG-1^−^CTLA-4^−^ from bulk CD8^+^ TILs. Furthermore, they found that a higher frequency of advanced differentiation stage T cells (CD27^−^CCR7^−^CD45RA^−^) among the shared subset (PD-1^+^Tim-3^+^TIGIT^+^2B4^+^KLRG-1^−^CTLA-4^−^) in bulk CD8^+^ TILs was associated with a poorly differentiated cancer type in cervical cancer patients.^152^ Similarly, PD-L1, PD-1, and HPV expression was more frequently seen as positive in cervical cancer tissues compared to normal cervical tissues, especially those strongly stained for HPV and stained in tissues from advanced tumor and tumor with lymphoid nodes or vascular invasion^153^ or tumor-lymph node-metastasis (TNM) stage.^154^ In addition, patients with a history of chemotherapy had an overexpression of PD-L1 in tumor cells and more PD-1 in stromal TILs,^153^ and patients with PD-L1^−^ and PD-1^+^ in TILs had poorer OS and disease-free survival.^154^^,^^155^ The above data indicate that PD-L1 can act as a biomarker in the worse prognosis and adverse clinicopathologic features of cervical cancer, and thus anti-PD-L1 therapy may be a useful approach in the treatment of cervical cancer patients.

Apart from the common PD-1/PD-L1, a study reported the uncommon PD-L2 in cervical cancer. Patients with SCC were sub-grouped into responder and non-responder according to standard chemoradiation therapy response. The responder patients exhibited an increase of an inflammatory TME as indicated by higher numbers of CD8^+^ and PD-L2^+^ TILs, as well as higher expression of a PD-L1 immunoreactive area, as compared to the non-responder group. Additionally, a correlation between the number of gene mutations and PD-L2^+^ TILs in the responder group was observed.^69^ This study indicates that PD-L2 may relate to neoantigens and needs to be considered for checkpoint therapy in cervical cancer. Further proving this, Feng et al.^156^ examined the deficient DNA mismatch repair (dMMR) and correlated dMMR status with PD-1/PD-L1 expression in cervical cancer patients. According to the expression of MLH1, MSH2, and the MSI (microsatellite instability) test, 66 patients were divided into dMMR or proficient DNA mismatch repair (pMMR) groups. PD-L1 in cancer cells, PD-L1 in TILs, and PD-1 in TILs took up 59.1%, 47.0%, and 60.6%, respectively. A statistical difference of PD-L1 in cancer cells could be observed between dMMR and pMMR subgroups. In the dMMR group, PD-L1 in cancer cells and PD-1 in TILs had no correlation (rs = 0.161, p = 0.537), but in the pMMR group, they had a good correlation (rs = 0.645, p < 0.001).

Despite the solid evidence mentioned above, a few studies have reported different results. For instance, a study reported that of 59 patients included, PD-L1 immunohistochemical expression was observed in only 32.2%. Among PD-L1 expression cases, only 8.5% showed strong-positive staining (>50%). However, there was no significant association between PD-L1 expression and clinicopathological features. The median PFS was 11.5 months for PD-L1-positive cases and 24.3 months for PD-L1-negative cases (p = 0.26). The median OS was 47.8 months for PD-L1-negative patients and was not reached for PD-L1-positive cases (p = 0.96). PD-L1 expression was observed in 27.1% of TILs and exerted no statistical impact on OS or PFS.^157^ From this study, it seems that PD-L1 is expressed in a minority of cervical cancer cases and is not related to clinicopathological characteristics or to survival outcomes in this cohort. Supporting this, Kawachi et al.^89^ analyzed TILs for PD-1 and tumors for PD-L1 in 148 patients with cervical adenocarcinoma and showed that most cases (54.7%) were classified in stage 1b and 26 (17.6%) had recurrent disease. The density of stromal infiltrating PD-1^+^ lymphocytes that had infiltrated invasive adenocarcinoma (n = 127) was significantly higher than in adenocarcinoma *in situ* (n = 27). However, expression of PD-L1 on tumor cells was found in 17.3% of invasive adenocarcinoma and was not associated with HPV infection status. A similar result was reported after an analysis of 219 cervical squamous cell cancers and 30 healthy controls.^154^ The summary from these different studies may suggest that the cohorts and studying methods need more detailed comparison to elucidate PD-L1 expression and function in cervical cancer, including its relationship with HPV infection.

#### Immune checkpoint therapy in cervical cancer

To prove the therapeutic value of PD-1/PD-L1 in cervical cancer, an immunotherapy against HPV16 genes E6 and E7 with adenoviral delivery vector (Ad5 [E1-, E2b-]-E6/E7) and a combination of PD-1 blockade was conducted. Ad5 [E1-, E2b-]-E6/E7 alone induced HPV E6/E7 cell-mediated immunity and resulted in the clearance of small tumors and an OS benefit in mice with larger established tumors. When the vaccination was combined with immune checkpoint blockade, an increased level of anti-tumor activity against large tumors was observed. Analysis of the TME in Ad5 [E1-, E2b-]-E6/E7-treated mice revealed elevated CD8^+^ TILs and the induction of suppressive mechanisms such as PD-L1 expression on tumor cells, and an increase in PD-1^+^ TILs was clear. When the treatment was combined with anti-PD-1 antibody, CD8^+^ TILs were at the same level, but a reduction in PD-L1 expression on tumor cells and reduced PD-1^+^ TILs were seen.^148^ This suggests that CD8^+^ TIL-mediated tumor calescence and a combination of immune checkpoint inhibition can further increase their killing ability.

In addition, a review article summarized immune checkpoint inhibitor (ICPI) treatment for different types of gynecological cancers by assessing the scanty data available from the literature and the perspective of clinical research ICPI therapies. The authors highlighted that the objective ICPI response rates ranged from 5.9% to 15% in early phase IB to II trials, including patients with platinum-resistant ovarian cancer, whereas only anecdotal data are available for patients with recurrent, heavily pre-treated endometrial cancer. They mentioned that several ongoing trials are investigating ICPI alone or in combination with chemotherapy or with other biological agents in untreated and recurrent ovarian cancer, advanced and recurrent endometrial cancer, as well as advanced and recurrent cervical cancer.^158^ Additionally, in another review article, the authors indicated that phase I/II clinical trials evaluating the effects of PD-1/PD-L1 targeted therapies were in progress for cervical carcinoma.^3^ All of these data support that inhibition of immune checkpoints such as PD-1 and its ligand PD-L1 may benefit immunotherapy of cervical cancer. We await the clinical trial results of these studies. However, so far there is no report on direct blockade of PD-1 of TILs during expansion or before infusion.

### Inhibition of immune-related signaling pathways

Tregs in TILs govern the cytolytic functions of CD8^+^ TILs through inhibitory signals/pathways. Understanding these molecules and pathways will facilitate the development of approaches to enhance the function of CD8^+^ TILs or other TILs such as NK and CD4^+^ cells. So far, there is limited information on this subject for cervical cancer. Some early evidence suggests that the expression levels of NKRs on CTLs are linked to their cytotoxicity function. A study examined the expression of various NKRs on TILs derived from cervical cancer and showed that the percentage expression of NKR^+^/CD8^+^ T lymphocytes was similar in gated CD8^+^ autologous TILs and PBMCs. On the contrary, CD8^+^ TILs expressed upregulated C-type lectin NKRs CD94/NKG2A compared with either peripheral blood CD8^+^ T cells or normal cervix-infiltrating CD8^+^ T lymphocytes.^159^ Dual NKR co-expression analyses showed that CD94 and NKG2A were mainly expressed on CD56^−^CD161^−^CD8^+^ TILs within the cancer milieu. IHC study showed that cervical cancer cells expressed abundant IL-15 and TGF-β. In a kinetic co-culture assay, cervical cancer cells can promote the expression of CD94/NKG2A on CD8^+^ T lymphocytes. The cancer-derived effects can be reversed by addition of rIL-15Rα/Fc and anti-TGF-β antibody. Functional analyses illustrated that intracellular perforin expression of CD8^+^ T cells was minimal upon upregulation of CD94/NKG2A. Kinetic cytotoxicity assays showed that upregulated expression of CD94/NKG2A restrained CD8^+^ T lymphocyte cytotoxicity.^159^ The data indicated that cervical cancer cells could promote the expression of inhibitory NKRs via an IL-15- and possibly TGF-β-mediated mechanism and abrogate the antitumor cytotoxicity of TILs. According to this study, the use of antibody to block IL-15 or inhibit the TGF-β pathway may enhance CD8^+^ TIL function.

### Enhancement with nanoparticles (NPs)

**Figure 4:**
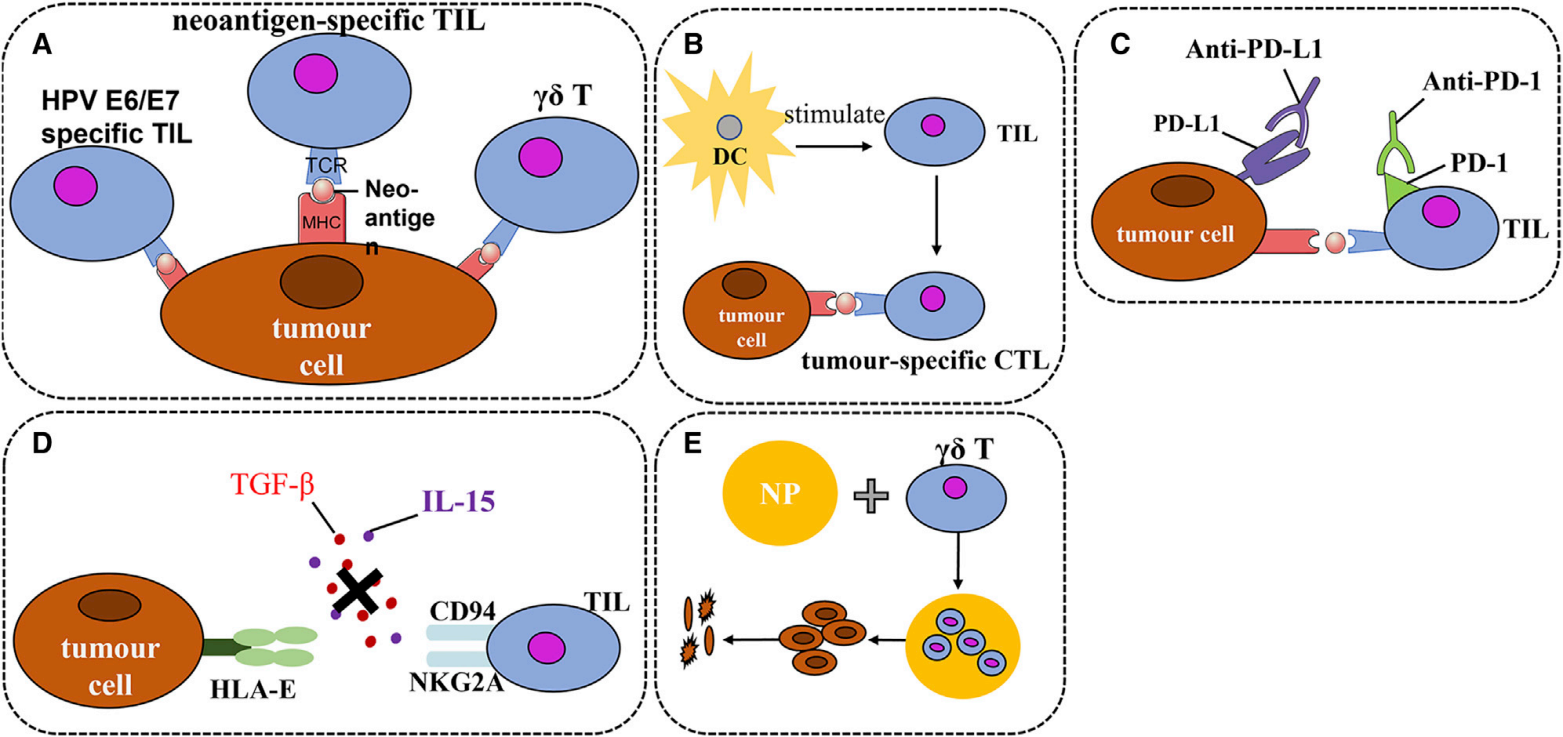
Methods to enhance therapeutic TILs (A–E) The cytotoxicity to tumors of therapeutic TILs can be enhanced via five methods, including selective expansion of HPV and/or neoantigen-reactive TILs (A), boosting TILs with an autogenetic DC vaccine (B), inhibiting negative checkpoints of TILs (C), inhibiting the function of Tregs (D), and enhancing the TIL killing function by special nanoparticles (NPs, E). Note that HLA-E, an MHC class Ib molecule, is a ligand for the inhibitory CD94/NKG2A receptor.^164^

A special and interesting way to promote TIL function is to employ NPs to enhance TIL function. A recent study reported the use of selenium NPs (SeNPs) to strengthen the anti-tumor cytotoxicity of Vγ9Vδ2 T cells, a subset of peripheral γδ T cells with promising anti-tumor activity. They showed that SeNP-pretreated γδ T cells had significantly stronger cancer killing and tumor growth inhibition efficacy, compared to γδ T cells alone. Furthermore, SeNP pretreatment could significantly upregulate the expression of cytotoxicity-related molecules, including NKG2D, CD16, and IFN-γ, and downregulate PD-1 expression of γδ T cells. Interestingly, the study found that SeNPs could promote tubulin acetylation in γδ T cells through interaction between the microtubule network and lysosomes.^160^ This work demonstrates a new strategy to further increase anti-tumor cytotoxicity of human γδ T cells by using an NP-based strategy. Whether this NP platform can be used in other T cells, especially TILs, requires further investigations. A summary of the above methods to enhance therapeutic TILs is illustrated in Figure 4.

## Future perspectives

The prognostic values of TILs in cervical cancer have been encouraging, and some of them will soon be applied to clinical practice, while more advances in therapeutic TILs are evidently expected.

### Selective expansion of specific TIL populations and combinational therapy

1) Tumor-specific TILs: TILs contain tumor-specific CTLs and CD4^+^ effectors, and their high numbers and ratio can be further optimized to kill tumor cells more precisely and effectively with reduced side effects. For viral-induced cancers such as cervical cancer, this can be achieved through selective expansion of viral antigen-specific TILs, such as HPV E6/E7. In addition, highly expressed neoantigens in cervical cancer, which may or may not be related to HPV, could be an option. As we understand more about cancer development and develop improve techniques to identify and isolate neoantigen-reactive TILs, we can more selectively expand neoantigen-specific TILs covering more heterogeneous tumor cell populations for better treatment outcomes.
2) Special TIL cell populations: T cells with certain TCRs represent a special population or are associated with unique functions, e.g., γδ T cells. Selection of these cell populations may also improve TIL therapy. In addition, further modification of their TCRs or targeting specificity such as the CAR-T technique could also be of interest in the future. NK cells have shown some exciting ability in treating other cancer patients. Future advancements of NK-based therapy for cervical cancer may be achieved by restoring their functionality *ex vivo.* The advances of single-cell technology will make featuring a single TIL cell by its gene and protein profiles possible. In this case, a T cell clone with anti-tumor profiles may be identified and expanded for specific TIL therapy, although this may be costly and time-consuming. Finally, as young TILs with CD27/CD28 biomarkers or CD8^+^ T cells with stemness^161^^,^^162^ represent an energetic and lasting TIL population, they have the potential to lead to better treatment outcomes. Future studies as well as clinical trials are expected to continue this trend.
3) Combinational TIL therapy: In the current regime of TIL therapy, a 1-week preparation treatment with cyclophosphamide or fludarabine is included. In addition, the therapy alone may not be sufficient to totally control the tumor growth, especially advanced tumors with a high tumor mass. In this case, combinational therapy with current approaches, such as chemotherapy or radiotherapy and surgery that can quickly reduce the tumor mass, may result in a better treatment outcome. More importantly, the combination with PD-1/PD-L1-based therapies will produce a synergistic treatment effect. Future efforts will emphasize how and when to logically and effectively combine the treatment regimen to maximize the treatment outcome.

### *In vitro* further priming

Although TILs contain a higher percentage of tumor-specific T cells, there is still a good portion of non-specific TILs. These cells can be further primed by *in vitro* procedures, or the activated TILs can be further boosted by tumor antigens. There have been some reports on using DC vaccines to prime or boost TILs.^147^^,^^148^ Future directions may focus on exploring what kind of antigen (including neoantigen) should be used to load onto DCs for boosting and the conditions of DCs for boosting such as the use of NPs and co-stimulatory factors to adjuvant DCs. In addition, cross-priming and presentation may be considered with MHC class I in the future to specifically target CD8^+^ T cells, as they are the major functional and anti-tumor T cells in TILs, while MHC class II should also be considered for CD4^+^ TIL priming and boosting.

### Inhibiting negative regulators

1) Blocking or suppressing negative checkpoints: TILs isolated from tumor tissue are heavily influenced by the TME that is composed of Tregs, TAMs, and inhibitory factors. Even after expansion, these negative regulators may still exist or are even highly expressed, such as checkpoint PD-1, and will exhaust TIL functions when transferred to patients. Suppressing these factors before infusion may be helpful for enhancing TIL function. Some researchers have already used PD-1 antibodies to treat TILs, and other methods, such as gene silencing of these checkpoints, may also be a promising way to increase cytotoxic TIL function.^151^ Using chemical inhibitors during *in vitro* expansion could be another alternative. There is no report on this at present, but it is expected soon. Besides PD-1, other immune checkpoints such as CLTA-4, KLAG-1, and TIM3 can also be considered in the future to further enhance TIL immune function.
2) Manipulating inhibitory factors from regulatory immune cells *in vitro* and *in vivo*: As described, TILs consist of different subsets of regulatory cells. However, after expansion the proportion of each subset may change but inhibitory T cells may remain, such as CD4^+^CD25^+^ T cells. They will play the negative role in the early stage of expansion and inhibit effector TIL function after infusion. Therefore, suppressing their function or blocking their inhibitory factors by antibodies can be necessary for enhancing or restoring effector TIL function. Besides *in vitro* approaches, *in vivo* preparation for TIL therapy is also a constructive approach for future TIL therapy. In particular, the use of PD-L1 antibody to block tumor cell checkpoints or antibodies against inhibitory factors such as IL-10 or TGF-β in the TME could be a useful way to indirectly increase the efficacy of TIL therapy. However, because of the complex of PD-L1 expression in tumor cells, how to choose a proper approach such as antibody blockade, gene silencing, and chemical inhibitors needs more careful examination of patient condition on a case-by-case basis.^6^ To achieve a targeted delivery of PD-L1 antibody to tumor cells or IL-10 antibody to TME, nanotechnology for drug delivery may be considered in the future.

### Enhancement of T cell functions in general

There is a consideration of increasing quality rather than quantity of TILs via employing other cytokines or NPs during TIL expansion. Increasing the cytotoxicity of TILs using NPs mentioned above (e.g., SeNPs) and other cytokines such as IL-12 during TIL *in vitro* expansion and even *in vivo* by intra-tumor injection^163^ to further increase the cytotoxicity of cytotoxic TILs could be an alternative to enhance the efficacy of TIL therapy.

## References

[1] Wang R., Pan W., Jin L., Huang W., Li Y., Wu D., Gao C., Ma D., Liao S. (2020). Human papillomavirus vaccine against cervical cancer: Opportunity and challenge. Cancer Lett..

[2] Hull R., Mbele M., Makhafola T., Hicks C., Wang S.M., Reis R.M., Mehrotra R., Mkhize-Kwitshana Z., Kibiki G., Bates D.O., Dlamini Z. (2020). Cervical cancer in low and middle-income countries. Oncol. Lett..

[3] Zhang L., Zhao Y., Tu Q., Xue X., Zhu X., Zhao K.N. (2021). The role of programmed cell death ligand-1/programmed cell death-1 (PD-L1/PD-1) in HPV-induced cervical cancer and potential for their use in blockade therapy. Curr. Med. Chem..

[4] Singh A.K., McGuirk J.P. (2020). CAR T cells: Continuation in a revolution of immunotherapy. Lancet Oncol..

[5] Hong M., Clubb J.D., Chen Y.Y. (2020). Engineering CAR-T cells for next-generation cancer therapy. Cancer Cell.

[6] Wu Y., Chen W., Xu Z.P., Gu W. (2019). PD-L1 distribution and perspective for cancer immunotherapy-blockade, knockdown, or inhibition. Front. Immunol..

[7] Gu W., Wang L., Wu Y., Liu J.P. (2019). Undo the brake of tumour immune tolerance with antibodies, peptide mimetics and small molecule compounds targeting PD-1/PD-L1 checkpoint at different locations for acceleration of cytotoxic immunity to cancer cells. Clin. Exp. Pharmacol. Physiol..

[8] Yang X., Wang G.X., Zhou J.F. (2019). CAR T cell therapy for hematological malignancies. Curr. Med. Sci..

[9] Leach D.R., Krummel M.F., Allison J.P. (1996). Enhancement of antitumor immunity by CTLA-4 blockade. Science.

[10] Kwon E.D., Hurwitz A.A., Foster B.A., Madias C., Feldhaus A.L., Greenberg N.M., Burg M.B., Allison J.P. (1997). Manipulation of T cell costimulatory and inhibitory signals for immunotherapy of prostate cancer. Proc. Natl. Acad. Sci. USA.

[11] Atkins M.B., Clark J.I., Quinn D.I. (2017). Immune checkpoint inhibitors in advanced renal cell carcinoma: Experience to date and future directions. Ann. Oncol..

[12] Hodi F.S., Mihm M.C., Soiffer R.J., Haluska F.G., Butler M., Seiden M.V., Davis T., Henry-Spires R., MacRae S., Willman A. (2003). Biologic activity of cytotoxic T lymphocyte-associated antigen 4 antibody blockade in previously vaccinated metastatic melanoma and ovarian carcinoma patients. Proc. Natl. Acad. Sci. USA.

[13] Gorabi A.M., Hajighasemi S., Sathyapalan T., Sahebkar A. (2020). Cell transfer-based immunotherapies in cancer: A review. IUBMB Life.

[14] Strizova Z., Bartunkova J., Smrz D. (2019). The challenges of adoptive cell transfer in the treatment of human renal cell carcinoma. Cancer Immunol. Immunother..

[15] Rosenberg S.A., Packard B.S., Aebersold P.M., Solomon D., Topalian S.L., Toy S.T., Simon P., Lotze M.T., Yang J.C., Seipp C.A. (1988). Use of tumor-infiltrating lymphocytes and interleukin-2 in the immunotherapy of patients with metastatic melanoma. A preliminary report. N. Engl. J. Med..

[16] Rosenberg S.A., Spiess P., Lafreniere R. (1986). A new approach to the adoptive immunotherapy of cancer with tumor-infiltrating lymphocytes. Science.

[17] Savas P., Salgado R., Denkert C., Sotiriou C., Darcy P.K., Smyth M.J., Loi S. (2016). Clinical relevance of host immunity in breast cancer: From TILs to the clinic. Nat. Rev. Clin. Oncol..

[18] Robbins P.F. (2017). Tumor-infiltrating lymphocyte therapy and neoantigens. Cancer J..

[19] Parkhurst M.R., Robbins P.F., Tran E., Prickett T.D., Gartner J.J., Jia L., Ivey G., Li Y.F., El-Gamil M., Lalani A. (2019). Unique neoantigens arise from somatic mutations in patients with gastrointestinal cancers. Cancer Discov..

[20] Yossef R., Tran E., Deniger D.C., Gros A., Pasetto A., Parkhurst M.R., Gartner J.J., Prickett T.D., Cafri G., Robbins P.F., Rosenberg S.A. (2018). Enhanced detection of neoantigen-reactive T cells targeting unique and shared oncogenes for personalized cancer immunotherapy. JCI Insight.

[21] Mantovani A., Allavena P., Sica A., Balkwill F. (2008). Cancer-related inflammation. Nature.

[22] Rollins B.J. (2006). Inflammatory chemokines in cancer growth and progression. Eur. J. Cancer.

[23] Moore O.S., Foote F.W. (1949). The relatively favorable prognosis of medullary carcinoma of the breast. Cancer.

[24] Clark W.H., From L., Bernardino E.A., Mihm M.C. (1969). The histogenesis and biologic behavior of primary human malignant melanomas of the skin. Cancer Res..

[25] Day C.L., Sober A.J., Kopf A.W., Lew R.A., Mihm M.C., Hennessey P., Golomb F.M., Harris M.N., Gumport S.L., Raker J.W. (1981). A prognostic model for clinical stage I melanoma of the upper extremity. The importance of anatomic subsites in predicting recurrent disease. Ann. Surg..

[26] Tuthill R.J., Unger J.M., Liu P.Y., Flaherty L.E., Sondak V.K., Southwest Oncology Group (2002). Risk assessment in localized primary cutaneous melanoma: A Southwest Oncology Group study evaluating nine factors and a test of the Clark logistic regression prediction model. Am. J. Clin. Pathol..

[27] Fefer A. (1969). Immunotherapy and chemotherapy of Moloney sarcoma virus-induced tumors in mice. Cancer Res..

[28] Eberlein T.J., Rosenstein M., Rosenberg S.A. (1982). Regression of a disseminated syngeneic solid tumor by systemic transfer of lymphoid cells expanded in interleukin 2. J. Exp. Med..

[29] Cheever M.A., Kempf R.A., Fefer A. (1977). Tumor neutralization, immunotherapy, and chemoimmmunotherapy of a Friend leukemia with cells secondarily sensitized in vitro. J. Immunol..

[30] Donohue J.H., Rosenstein M., Chang A.E., Lotze M.T., Robb R.J., Rosenberg S.A. (1984). The systemic administration of purified interleukin 2 enhances the ability of sensitized murine lymphocytes to cure a disseminated syngeneic lymphoma. J. Immunol..

[31] Rosenberg S.A., Yannelli J.R., Yang J.C., Topalian S.L., Schwartzentruber D.J., Weber J.S., Parkinson D.R., Seipp C.A., Einhorn J.H., White D.E. (1994). Treatment of patients with metastatic melanoma with autologous tumor-infiltrating lymphocytes and interleukin 2. J. Natl. Cancer Inst..

[32] Dudley M.E., Gross C.A., Langhan M.M., Garcia M.R., Sherry R.M., Yang J.C., Phan G.Q., Kammula U.S., Hughes M.S., Citrin D.E. (2010). CD8+ enriched “young” tumor infiltrating lymphocytes can mediate regression of metastatic melanoma. Clin. Cancer Res..

[33] Rosenberg S.A., Yang J.C., Sherry R.M., Kammula U.S., Hughes M.S., Phan G.Q., Citrin D.E., Restifo N.P., Robbins P.F., Wunderlich J.R. (2011). Durable complete responses in heavily pretreated patients with metastatic melanoma using T-cell transfer immunotherapy. Clin. Cancer Res..

[34] Goff S.L., Dudley M.E., Citrin D.E., Somerville R.P., Wunderlich J.R., Danforth D.N., Zlott D.A., Yang J.C., Sherry R.M., Kammula U.S. (2016). Randomized, prospective evaluation comparing intensity of lymphodepletion before adoptive transfer of tumor-infiltrating lymphocytes for patients with metastatic melanoma. J. Clin. Oncol..

[35] Muller A.J., Scherle P.A. (2006). Targeting the mechanisms of tumoral immune tolerance with small-molecule inhibitors. Nat. Rev. Cancer.

[36] Khosravi N., Mokhtarzadeh A., Baghbanzadeh A., Hajiasgharzadeh K., Shahgoli V.K., Hemmat N., Safarzadeh E., Baradaran B. (2020). Immune checkpoints in tumor microenvironment and their relevance to the development of cancer stem cells. Life Sci..

[37] Dieci M.V., Radosevic-Robin N., Fineberg S., van den Eynden G., Ternes N., Penault-Llorca F., Pruneri G., D’Alfonso T.M., Demaria S., Castaneda C. (2018). Update on tumor-infiltrating lymphocytes (TILs) in breast cancer, including recommendations to assess TILs in residual disease after neoadjuvant therapy and in carcinoma in situ: A report of the International Immuno-Oncology Biomarker Working Group on Breast Cancer. Semin. Cancer Biol..

[38] Drake C.G., Jaffee E., Pardoll D.M. (2006). Mechanisms of immune evasion by tumors. Adv. Immunol..

[39] Pfizenmaier K., Scheurich P., Schlüter C., Krönke M. (1987). Tumor necrosis factor enhances HLA-A,B,C and HLA-DR gene expression in human tumor cells. J. Immunol.

[40] Ruiter D.J., Mattijssen V., Broecker E.B., Ferrone S. (1991). MHC antigens in human melanomas. Semin. Cancer Biol..

[41] Bröcker E.B., Suter L., Sorg C. (1984). HLA-DR antigen expression in primary melanomas of the skin. J. Invest. Dermatol..

[42] Baton F., Deruyffelaere C., Chapin M., Prod’homme T., Charron D., Al-Daccak R., Alcaide-Loridan C. (2004). Class II transactivator (CIITA) isoform expression and activity in melanoma. Melanoma Res..

[43] Cheng W.-F., Lee C.-N., Chang M.-C., Su Y.-N., Chen C.-A., Hsieh C.-Y. (2005). Antigen-specific CD8^+^ T lymphocytes generated from a DNA vaccine control tumors through the Fas-FasL pathway. Mol. Ther..

[44] Ivanova O.K., Sharapova T.N., Romanova E.A., Soshnikova N.V., Sashchenko L.P., Yashin D.V. (2017). CD3^+^ CD8^+^ NKG2D^+^ T lymphocytes induce apoptosis and necroptosis in HLA-negative cells via FasL-Fas interaction. J. Cell. Biochem..

[45] Masopust D., Schenkel J.M. (2013). The integration of T cell migration, differentiation and function. Nat. Rev. Immunol..

[46] Schenkel J.M., Masopust D. (2014). Tissue-resident memory T cells. Immunity.

[47] Mackay L.K., Rahimpour A., Ma J.Z., Collins N., Stock A.T., Hafon M.L., Vega-Ramos J., Lauzurica P., Mueller S.N., Stefanovic T. (2013). The developmental pathway for CD103^+^CD8^+^ tissue-resident memory T cells of skin. Nat. Immunol..

[48] Sathaliyawala T., Kubota M., Yudanin N., Turner D., Camp P., Thome J.J., Bickham K.L., Lerner H., Goldstein M., Sykes M. (2013). Distribution and compartmentalization of human circulating and tissue-resident memory T cell subsets. Immunity.

[49] Gagliani N., Amezcua Vesely M.C., Iseppon A., Brockmann L., Xu H., Palm N.W., de Zoete M.R., Licona-Limón P., Paiva R.S., Ching T. (2015). Th17 cells transdifferentiate into regulatory T cells during resolution of inflammation. Nature.

[50] Downs-Canner S., Berkey S., Delgoffe G.M., Edwards R.P., Curiel T., Odunsi K., Bartlett D.L., Obermajer N. (2017). Suppressive IL-17A^+^Foxp3^+^ and ex-Th17 IL-17A^neg^Foxp3^+^ T_reg_ cells are a source of tumour-associated T_reg_ cells. Nat. Commun..

[51] Zhou J., Shen X., Huang J., Hodes R.J., Rosenberg S.A., Robbins P.F. (2005). Telomere length of transferred lymphocytes correlates with in vivo persistence and tumor regression in melanoma patients receiving cell transfer therapy. J. Immunol.

[52] Berger C., Jensen M.C., Lansdorp P.M., Gough M., Elliott C., Riddell S.R. (2008). Adoptive transfer of effector CD8^+^ T cells derived from central memory cells establishes persistent T cell memory in primates. J. Clin. Invest..

[53] Robbins P.F., Dudley M.E., Wunderlich J., El-Gamil M., Li Y.F., Zhou J., Huang J., Powell D.J., Rosenberg S.A. (2004). Cutting edge: Persistence of transferred lymphocyte clonotypes correlates with cancer regression in patients receiving cell transfer therapy. J. Immunol..

[54] Donia M., Junker N., Ellebaek E., Andersen M.H., Straten P.T., Svane I.M. (2012). Characterization and comparison of “standard” and “young” tumour-infiltrating lymphocytes for adoptive cell therapy at a Danish translational research institution. Scand. J. Immunol..

[55] Chacon J.A., Wu R.C., Sukhumalchandra P., Molldrem J.J., Sarnaik A., Pilon-Thomas S., Weber J., Hwu P., Radvanyi L. (2013). Co-stimulation through 4-1BB/CD137 improves the expansion and function of CD8^+^ melanoma tumor-infiltrating lymphocytes for adoptive T-cell therapy. PLoS ONE.

[56] Li Y., Bleakley M., Yee C. (2005). IL-21 influences the frequency, phenotype, and affinity of the antigen-specific CD8 T cell response. J. Immunol..

[57] Hinrichs C.S., Spolski R., Paulos C.M., Gattinoni L., Kerstann K.W., Palmer D.C., Klebanoff C.A., Rosenberg S.A., Leonard W.J., Restifo N.P. (2008). IL-2 and IL-21 confer opposing differentiation programs to CD8^+^ T cells for adoptive immunotherapy. Blood.

[58] Heaton K.M., Ju G., Grimm E.A. (1993). Human interleukin 2 analogues that preferentially bind the intermediate-affinity interleukin 2 receptor lead to reduced secondary cytokine secretion: Implications for the use of these interleukin 2 analogues in cancer immunotherapy. Cancer Res..

[59] Heaton K.M., Ju G., Grimm E.A. (1994). Induction of lymphokine-activated killing with reduced secretion of interleukin-1β, tumor necrosis factor-α, and interferon-γ by interleukin-2 analogs. Ann. Surg. Oncol..

[60] Sim G.C., Martin-Orozco N., Jin L., Yang Y., Wu S., Washington E., Sanders D., Lacey C., Wang Y., Vence L. (2014). IL-2 therapy promotes suppressive ICOS^+^ Treg expansion in melanoma patients. J. Clin. Invest..

[61] Maskey N., Thapa N., Maharjan M., Shrestha G., Maharjan N., Cai H., Liu S. (2019). Infiltrating CD4 and CD8 lymphocytes in HPV infected uterine cervical milieu. Cancer Manag. Res..

[62] Bedoya A.M., Jaramillo R., Baena A., Castaño J., Olaya N., Zea A.H., Herrero R., Sanchez G.I. (2013). Location and density of immune cells in precursor lesions and cervical cancer. Cancer Microenviron..

[63] Etxeberria I., Bolaños E., Quetglas J.I., Gros A., Villanueva A., Palomero J., Sánchez-Paulete A.R., Piulats J.M., Matias-Guiu X., Olivera I. (2019). Intratumor adoptive transfer of IL-12 mRNA transiently engineered antitumor CD8^+^ T cells. Cancer Cell.

[64] Bell M.C., Edwards R.P., Partridge E.E., Kuykendall K., Conner W., Gore H., Turbat-Herrara E., Crowley-Nowick P.A. (1995). CD8^+^ T lymphocytes are recruited to neoplastic cervix. J. Clin. Immunol..

[65] Gooden M.J., de Bock G.H., Leffers N., Daemen T., Nijman H.W. (2011). The prognostic influence of tumour-infiltrating lymphocytes in cancer: A systematic review with meta-analysis. Br. J. Cancer.

[66] Shah W., Yan X., Jing L., Zhou Y., Chen H., Wang Y. (2011). A reversed CD4/CD8 ratio of tumor-infiltrating lymphocytes and a high percentage of CD4^+^FOXP3^+^ regulatory T cells are significantly associated with clinical outcome in squamous cell carcinoma of the cervix. Cell. Mol. Immunol..

[67] Sheu B.C., Hsu S.M., Ho H.N., Lin R.H., Torng P.L., Huang S.C. (1999). Reversed CD4/CD8 ratios of tumor-infiltrating lymphocytes are correlated with the progression of human cervical carcinoma. Cancer.

[68] Wang J., Li Z., Gao A., Wen Q., Sun Y. (2019). The prognostic landscape of tumor-infiltrating immune cells in cervical cancer. Biomed. Pharmacother..

[69] Martins P.R., Machado C.M.T., Coxir S.A., de Oliveira A.J., Moreira T.B., Campos L.S., Alcântara R., de Paula S.O.C., de Oliveira Salles P.G., Gollob K.J., Magalhães W.C.S. (2019). Cervical cancer patients that respond to chemoradiation therapy display an intense tumor infiltrating immune profile before treatment. Exp. Mol. Pathol..

[70] Heeren A.M., van Luijk I.F., Lakeman J., Pocorni N., Kole J., de Menezes R.X., Kenter G.G., Bosse T., de Kroon C.D., Jordanova E.S. (2019). Neoadjuvant cisplatin and paclitaxel modulate tumor-infiltrating T cells in patients with cervical cancer. Cancer Immunol. Immunother..

[71] Dorta-Estremera S., Colbert L.E., Nookala S.S., Yanamandra A.V., Yang G., Delgado A., Mikkelson M., Eifel P., Jhingran A., Lilie L.L. (2018). Kinetics of intratumoral immune cell activation during chemoradiation for cervical cancer. Int. J. Radiat. Oncol. Biol. Phys..

[72] Miyasaka Y., Yoshimoto Y., Murata K., Noda S.E., Ando K., Ebara T., Okonogi N., Kaminuma T., Yamada S., Ikota H. (2020). Treatment outcomes of patients with adenocarcinoma of the uterine cervix after definitive radiotherapy and the prognostic impact of tumor-infiltrating CD8^+^ lymphocytes in pre-treatment biopsy specimens: a multi-institutional retrospective study. J. Radiat. Res. (Tokyo).

[73] Guillerey C., Huntington N.D., Smyth M.J. (2016). Targeting natural killer cells in cancer immunotherapy. Nat. Immunol..

[74] Hu W., Wang G., Huang D., Sui M., Xu Y. (2019). Cancer immunotherapy based on natural killer cells: Current progress and new opportunities. Front. Immunol..

[75] Sheu B.C., Lin R.H., Ho H.N., Huang S.C. (1997). Down-regulation of CD25 expression on the surface of activated tumor-infiltrating lymphocytes in human cervical carcinoma. Hum. Immunol..

[76] Chang W.C., Li C.H., Chu L.H., Huang P.S., Sheu B.C., Huang S.C. (2016). Regulatory T cells suppress natural killer cell immunity in patients with human cervical carcinoma. Int. J. Gynecol. Cancer.

[77] Kono K., Ressing M.E., Brandt R.M., Melief C.J., Potkul R.K., Andersson B., Petersson M., Kast W.M., Kiessling R. (1996). Decreased expression of signal-transducing zeta chain in peripheral T cells and natural killer cells in patients with cervical cancer. Clin. Cancer Res..

[78] Wu M.Y., Kuo T.Y., Ho H.N. (2011). Tumor-infiltrating lymphocytes contain a higher proportion of FOXP3^+^ T lymphocytes in cervical cancer. J. Formos. Med. Assoc..

[79] Adurthi S., Mukherjee G., Krishnamurthy H., Sudhir K., Bafna U.D., Umadevi K., Jayshree R.S. (2012). Functional tumor infiltrating T_H_1 and T_H_2 effectors in large early-stage cervical cancer are suppressed by regulatory T cells. Int. J. Gynecol. Cancer.

[80] Cao M., Wang Y., Wang D., Duan Y., Hong W., Zhang N., Shah W., Wang Y., Chen H. (2020). Increased high-risk human papillomavirus viral load is associated with immunosuppressed microenvironment and predicts a worse long-term survival in cervical cancer patients. Am. J. Clin. Pathol..

[81] Alves J.J.P., De Medeiros Fernandes T.A.A., De Araújo J.M.G., Cobucci R.N.O., Lanza D.C.F., Bezerra F.L., Andrade V.S., Fernandes J.V. (2018). Th17 response in patients with cervical cancer. Oncol. Lett..

[82] Hou F., Li Z., Ma D., Zhang W., Zhang Y., Zhang T., Kong B., Cui B. (2012). Distribution of Th17 cells and Foxp3-expressing T cells in tumor-infiltrating lymphocytes in patients with uterine cervical cancer. Clin. Chim. Acta.

[83] Punt S., van Vliet M.E., Spaans V.M., de Kroon C.D., Fleuren G.J., Gorter A., Jordanova E.S. (2015). FoxP3^+^ and IL-17^+^ cells are correlated with improved prognosis in cervical adenocarcinoma. Cancer Immunol. Immunother..

[84] Wu Y., Ye S., Goswami S., Pei X., Xiang L., Zhang X., Yang H. (2020). Clinical significance of peripheral blood and tumor tissue lymphocyte subsets in cervical cancer patients. BMC Cancer.

[85] Li H., Wang Y., Zhou F. (2010). Effect of ex vivo-expanded γδ-T cells combined with galectin-1 antibody on the growth of human cervical cancer xenografts in SCID mice. Clin. Invest. Med..

[86] Chanmee T., Ontong P., Konno K., Itano N. (2014). Tumor-associated macrophages as major players in the tumor microenvironment. Cancers (Basel).

[87] Quatromoni J.G., Eruslanov E. (2012). Tumor-associated macrophages: Function, phenotype, and link to prognosis in human lung cancer. Am. J. Transl. Res..

[88] Solinas G., Germano G., Mantovani A., Allavena P. (2009). Tumor-associated macrophages (TAM) as major players of the cancer-related inflammation. J. Leukoc. Biol..

[89] Kawachi A., Yoshida H., Kitano S., Hiraoka N. (2016). Tumor-infiltrating CD204+ M2 macrophages compared to T cells as a prognosticator for patients with uterine cervical adenocarcinoma. J. Clin. Oncol..

[90] Das D., Sarkar B., Mukhopadhyay S., Banerjee C., Biswas Mondal S. (2018). An altered ratio of CD4^+^ and CD8^+^ T lymphocytes in cervical cancer tissues and peripheral blood—A prognostic clue?. Asian Pac. J. Cancer Prev..

[91] Liang Y., Lü W., Zhang X., Lü B. (2018). Tumor-infiltrating CD8^+^ and FOXP3^+^ lymphocytes before and after neoadjuvant chemotherapy in cervical cancer. Diagn. Pathol..

[92] Jordanova E.S., Gorter A., Ayachi O., Prins F., Durrant L.G., Kenter G.G., van der Burg S.H., Fleuren G.J. (2008). Human leukocyte antigen class I, MHC class I chain-related molecule A, and CD8^+^/regulatory T-cell ratio: which variable determines survival of cervical cancer patients?. Clin. Cancer Res..

[93] Dudley M.E., Wunderlich J.R., Shelton T.E., Even J., Rosenberg S.A. (2003). Generation of tumor-infiltrating lymphocyte cultures for use in adoptive transfer therapy for melanoma patients. J. Immunol..

[94] Stevanović S., Draper L.M., Langhan M.M., Campbell T.E., Kwong M.L., Wunderlich J.R., Dudley M.E., Yang J.C., Sherry R.M., Kammula U.S. (2015). Complete regression of metastatic cervical cancer after treatment with human papillomavirus-targeted tumor-infiltrating T cells. J. Clin. Oncol..

[95] Stevanović S., Helman S.R., Wunderlich J.R., Langhan M.M., Doran S.L., Kwong M.L.M., Somerville R.P.T., Klebanoff C.A., Kammula U.S., Sherry R.M. (2019). A phase II study of tumor-infiltrating lymphocyte therapy for human papillomavirus-associated epithelial cancers. Clin. Cancer Res..

[96] Oerke S., Höhn H., Zehbe I., Pilch H., Schicketanz K.H., Hitzler W.E., Neukirch C., Freitag K., Maeurer M.J. (2005). Naturally processed and HLA-B8-presented HPV16 E7 epitope recognized by T cells from patients with cervical cancer. Int. J. Cancer.

[97] Zehbe I., Kaufmann A.M., Schmidt M., Hohn H., Maeurer M.J. (2007). Human papillomavirus 16 E6-specific CD45RA^+^ CCR7^+^ high avidity CD8^+^ T cells fail to control tumor growth despite interferon-gamma production in patients with cervical cancer. J. Immunother..

[98] Piersma S.J., Welters M.J., van der Hulst J.M., Kloth J.N., Kwappenberg K.M., Trimbos B.J., Melief C.J., Hellebrekers B.W., Fleuren G.J., Kenter G.G. (2008). Human papilloma virus specific T cells infiltrating cervical cancer and draining lymph nodes show remarkably frequent use of HLA-DQ and -DP as a restriction element. Int. J. Cancer.

[99] Hilders C.G., Ras L., van Eendenburg J.D., Nooyen Y., Fleuren G.J. (1994). Isolation and characterization of tumor-infiltrating lymphocytes from cervical carcinoma. Int. J. Cancer.

[100] Helman S.R., Stevanovic S., Campbell T.E., Kwong M.L.M., Doran S.L., Faquin W.C., Hinrichs C.S. (2018). Human papillomavirus T-cell cross-reactivity in cervical cancer: Implications for immunotherapy clinical trial design. JAMA Netw. Open.

[101] van Poelgeest M.I., Visconti V.V., Aghai Z., van Ham V.J., Heusinkveld M., Zandvliet M.L., Valentijn A.R., Goedemans R., van der Minne C.E., Verdegaal E.M. (2016). Potential use of lymph node-derived HPV-specific T cells for adoptive cell therapy of cervical cancer. Cancer Immunol. Immunother..

[102] Jin B.Y., Campbell T.E., Draper L.M., Stevanović S., Weissbrich B., Yu Z., Restifo N.P., Rosenberg S.A., Trimble C.L., Hinrichs C.S. (2018). Engineered T cells targeting E7 mediate regression of human papillomavirus cancers in a murine model. JCI Insight.

[103] Ye F., Chen H., Liang Z., Lu W., Cheng Q., Xie X. (2006). Establishment of a cervical cancer model via inoculating SiHa cells into humanized severe combined immunodeficient mice. Eur. J. Gynaecol. Oncol..

[104] Labarrière N., Pandolfino M.-C., Gervois N., Khammari A., Tessier M.-H., Dréno B., Jotereau F. (2002). Therapeutic efficacy of melanoma-reactive TIL injected in stage III melanoma patients. Cancer Immunol. Immunother..

[105] Andersen R., Donia M., Ellebaek E., Borch T.H., Kongsted P., Iversen T.Z., Hölmich L.R., Hendel H.W., Met Ö., Andersen M.H. (2016). Long-lasting complete responses in patients with metastatic melanoma after adoptive cell therapy with tumor-infiltrating lymphocytes and an attenuated IL2 regimen. Clin. Cancer Res..

[106] Besser M.J., Shapira-Frommer R., Itzhaki O., Treves A.J., Zippel D.B., Levy D., Kubi A., Shoshani N., Zikich D., Ohayon Y. (2013). Adoptive transfer of tumor-infiltrating lymphocytes in patients with metastatic melanoma: intent-to-treat analysis and efficacy after failure to prior immunotherapies. Clin. Cancer Res..

[107] Saint-Jean M., Knol A.-C., Volteau C., Quéreux G., Peuvrel L., Brocard A., Pandolfino M.-C., Saiagh S., Nguyen J.-M., Bedane C. (2018). Adoptive cell therapy with tumor-infiltrating lymphocytes in advanced melanoma patients. J. Immunol. Res..

[108] Pilon-Thomas S., Kuhn L., Ellwanger S., Janssen W., Royster E., Marzban S., Kudchadkar R., Zager J., Gibney G., Sondak V.K. (2012). Brief communication: Efficacy of adoptive cell transfer of tumor infiltrating lymphocytes after lymphopenia induction for metastatic melanoma. J. Immunother..

[109] Radvanyi L.G., Bernatchez C., Zhang M., Fox P.S., Miller P., Chacon J., Wu R., Lizee G., Mahoney S., Alvarado G. (2012). Specific lymphocyte subsets predict response to adoptive cell therapy using expanded autologous tumor-infiltrating lymphocytes in metastatic melanoma patients. Clin. Cancer Res..

[110] Nguyen L.T., Saibil S.D., Sotov V., Le M.X., Khoja L., Ghazarian D., Bonilla L., Majeed H., Hogg D., Joshua A.M. (2019). Phase II clinical trial of adoptive cell therapy for patients with metastatic melanoma with autologous tumor-infiltrating lymphocytes and low-dose interleukin-2. Cancer Immunol. Immunother..

[111] Kradin R.L., Kurnick J.T., Lazarus D.S., Preffer F.I., Dubinett S.M., Pinto C.E., Gifford J., Davidson E., Grove B., Callahan R.J. (1989). Tumour-infiltrating lymphocytes and interleukin-2 in treatment of advanced cancer. Lancet.

[112] Aoki Y., Takakuwa K., Kodama S., Tanaka K., Takahashi M., Tokunaga A., Takahashi T. (1991). Use of adoptive transfer of tumor-infiltrating lymphocytes alone or in combination with cisplatin-containing chemotherapy in patients with epithelial ovarian cancer. Cancer Res..

[113] Freedman R.S., Edwards C.L., Kavanagh J.J., Kudelka A.P., Katz R.L., Carrasco C.H., Atkinson E.N., Scott W., Tomasovic B., Templin S. (1994). Intraperitoneal adoptive immunotherapy of ovarian carcinoma with tumor-infiltrating lymphocytes and low-dose recombinant interleukin-2: A pilot trial. J. Immunother. Emphasis Tumor Immunol..

[114] Jiang S.-S., Tang Y., Zhang Y.-J., Weng D.-S., Zhou Z.-G., Pan K., Pan Q.-Z., Wang Q.-J., Liu Q., He J. (2015). A phase I clinical trial utilizing autologous tumor-infiltrating lymphocytes in patients with primary hepatocellular carcinoma. Oncotarget.

[115] Besser M.J., Shapira-Frommer R., Treves A.J., Zippel D., Itzhaki O., Schallmach E., Kubi A., Shalmon B., Hardan I., Catane R. (2009). Minimally cultured or selected autologous tumor-infiltrating lymphocytes after a lympho-depleting chemotherapy regimen in metastatic melanoma patients. J. Immunother..

[116] Itzhaki O., Hovav E., Ziporen Y., Levy D., Kubi A., Zikich D., Hershkovitz L., Treves A.J., Shalmon B., Zippel D. (2011). Establishment and large-scale expansion of minimally cultured “young” tumor infiltrating lymphocytes for adoptive transfer therapy. J. Immunother..

[117] Topalian S.L., Muul L.M., Solomon D., Rosenberg S.A. (1987). Expansion of human tumor infiltrating lymphocytes for use in immunotherapy trials. J. Immunol. Methods.

[118] Topalian S.L., Solomon D., Rosenberg S.A. (1989). Tumor-specific cytolysis by lymphocytes infiltrating human melanomas. J. Immunol..

[119] Tran K.Q., Zhou J., Durflinger K.H., Langhan M.M., Shelton T.E., Wunderlich J.R., Robbins P.F., Rosenberg S.A., Dudley M.E. (2008). Minimally cultured tumor-infiltrating lymphocytes display optimal characteristics for adoptive cell therapy. J. Immunother..

[120] Goff S.L., Smith F.O., Klapper J.A., Sherry R., Wunderlich J.R., Steinberg S.M., White D., Rosenberg S.A., Dudley M.E., Yang J.C. (2010). Tumor infiltrating lymphocyte therapy for metastatic melanoma: Analysis of tumors resected for TIL. J. Immunother..

[121] Joseph R.W., Peddareddigari V.R., Liu P., Miller P.W., Overwijk W.W., Bekele N.B., Ross M.I., Lee J.E., Gershenwald J.E., Lucci A. (2011). Impact of clinical and pathologic features on tumor-infiltrating lymphocyte expansion from surgically excised melanoma metastases for adoptive T-cell therapy. Clin. Cancer Res..

[122] Ben-Avi R., Itzhaki O., Simansky D., Zippel D., Markel G., Ben Nun A., Schachter J., Besser M.J. (2016). Metastatic lung lesions as a preferred resection site for immunotherapy with tumor infiltrating lymphocytes. J. Immunother..

[123] Nielsen M., Krarup-Hansen A., Hovgaard D., Petersen M.M., Loya A.C., Westergaard M.C.W., Svane I.M., Junker N. (2020). In vitro 4-1BB stimulation promotes expansion of CD8^+^ tumor-infiltrating lymphocytes from various sarcoma subtypes. Cancer Immunol. Immunother..

[124] Sakellariou-Thompson D., Forget M.-A., Creasy C., Bernard V., Zhao L., Kim Y.U., Hurd M.W., Uraoka N., Parra E.R., Kang Y. (2017). 4-1BB agonist focuses CD8^+^ tumor-infiltrating T-cell growth into a distinct repertoire capable of tumor recognition in pancreatic cancer. Clin. Cancer Res..

[125] Dudley M.E., Wunderlich J.R., Yang J.C., Sherry R.M., Topalian S.L., Restifo N.P., Royal R.E., Kammula U., White D.E., Mavroukakis S.A. (2005). Adoptive cell transfer therapy following non-myeloablative but lymphodepleting chemotherapy for the treatment of patients with refractory metastatic melanoma. J. Clin. Oncol..

[126] Yeh S., Karne N.K., Kerkar S.P., Heller C.K., Palmer D.C., Johnson L.A., Li Z., Bishop R.J., Wong W.T., Sherry R.M. (2009). Ocular and systemic autoimmunity after successful tumor-infiltrating lymphocyte immunotherapy for recurrent, metastatic melanoma. Ophthalmology.

[127] Chandran S.S., Somerville R.P.T., Yang J.C., Sherry R.M., Klebanoff C.A., Goff S.L., Wunderlich J.R., Danforth D.N., Zlott D., Paria B.C. (2017). Treatment of metastatic uveal melanoma with adoptive transfer of tumour-infiltrating lymphocytes: A single-centre, two-stage, single-arm, phase 2 study. Lancet Oncol..

[129] Zhang Y., Li S., Lai B., Wang H., Zhan X., Liu G., Wang Y. (2001). [Clinical research on the antitumor activity and phenotype of tumor infiltrating lymphocytes for treatment of lung cancer]. Zhongguo Fei Ai Za Zhi.

[130] Freedman R.S., Kudelka A.P., Kavanagh J.J., Verschraegen C., Edwards C.L., Nash M., Levy L., Atkinson E.N., Zhang H.Z., Melichar B. (2000). Clinical and biological effects of intraperitoneal injections of recombinant interferon-gamma and recombinant interleukin 2 with or without tumor-infiltrating lymphocytes in patients with ovarian or peritoneal carcinoma. Clin. Cancer Res..

[131] Eberlein T.J., Schoof D.D. (1991). The role of interleukin-2 in cancer immunotherapy. Compr. Ther..

[132] Rosenstein M., Ettinghausen S.E., Rosenberg S.A. (1986). Extravasation of intravascular fluid mediated by the systemic administration of recombinant interleukin 2. J. Immunol..

[133] Panelli M.C., White R., Foster M., Martin B., Wang E., Smith K., Marincola F.M. (2004). Forecasting the cytokine storm following systemic interleukin (IL)-2 administration. J. Transl. Med..

[134] Lotze M.T., Matory Y.L., Rayner A.A., Ettinghausen S.E., Vetto J.T., Seipp C.A., Rosenberg S.A. (1986). Clinical effects and toxicity of interleukin-2 in patients with cancer. Cancer.

[135] Boyman O., Sprent J. (2012). The role of interleukin-2 during homeostasis and activation of the immune system. Nat. Rev. Immunol..

[136] Boyman O., Surh C.D., Sprent J. (2006). Potential use of IL-2/anti-IL-2 antibody immune complexes for the treatment of cancer and autoimmune disease. Expert Opin. Biol. Ther..

[137] Kruit W.H., Punt K.J., Goey S.H., de Mulder P.H., van Hoogenhuyze D.C., Henzen-Logmans S.C., Stoter G. (1994). Cardiotoxicity as a dose-limiting factor in a schedule of high dose bolus therapy with interleukin-2 and alpha-interferon. An unexpectedly frequent complication. Cancer.

[138] Klempner M.S., Noring R., Mier J.W., Atkins M.B. (1990). An acquired chemotactic defect in neutrophils from patients receiving interleukin-2 immunotherapy. N. Engl. J. Med..

[139] Dhupkar P., Gordon N. (2017). Interleukin-2: Old and new approaches to enhance immune-therapeutic efficacy. Adv. Exp. Med. Biol..

[140] Teo P.Y., Yang C., Whilding L.M., Parente-Pereira A.C., Maher J., George A.J., Hedrick J.L., Yang Y.Y., Ghaem-Maghami S. (2015). Ovarian cancer immunotherapy using PD-L1 siRNA targeted delivery from folic acid-functionalized polyethylenimine: Strategies to enhance T cell killing. Adv. Healthc. Mater..

[141] Schumacher T.N., Scheper W., Kvistborg P. (2019). Cancer neoantigens. Annu. Rev. Immunol..

[142] Linette G.P., Carreno B.M. (2019). Tumor-infiltrating lymphocytes in the checkpoint inhibitor era. Curr. Hematol. Malig. Rep..

[143] Mathieu M., Paradis A., Pelletier S., Hébert S., Boutin K., Audemard É., Mader S., Kleinman C., Turcotte S. (2018). Evidence of neoantigen-reactive T cell response in a case of relapsing, mismatch-repair gene proficient, colorectal cancer. Cancer Res..

[144] Parkhurst M., Gros A., Pasetto A., Prickett T., Crystal J.S., Robbins P., Rosenberg S.A. (2017). Isolation of T-cell receptors specifically reactive with mutated tumor-associated antigens from tumor-infiltrating lymphocytes based on CD137 expression. Clin. Cancer Res..

[145] Li Q., Ding Z.Y. (2020). The ways of isolating neoantigen-specific T cells. Front. Oncol..

[146] Lu Y.C., Zheng Z., Robbins P.F., Tran E., Prickett T.D., Gartner J.J., Li Y.F., Ray S., Franco Z., Bliskovsky V. (2018). An efficient single-cell RNA-seq approach to identify neoantigen-specific T cell receptors. Mol. Ther..

[147] Santin A.D., Bellone S., Palmieri M., Bossini B., Roman J.J., Cannon M.J., Bignotti E., Canè S., Pecorelli S. (2003). Induction of tumor-specific cytotoxicity in tumor infiltrating lymphocytes by HPV16 and HPV18 E7-pulsed autologous dendritic cells in patients with cancer of the uterine cervix. Gynecol. Oncol..

[148] Rice A.E., Latchman Y.E., Balint J.P., Lee J.H., Gabitzsch E.S., Jones F.R. (2015). An HPV-E6/E7 immunotherapy plus PD-1 checkpoint inhibition results in tumor regression and reduction in PD-L1 expression. Cancer Gene Ther..

[149] Xin Yu J., Hodge J.P., Oliva C., Neftelinov S.T., Hubbard-Lucey V.M., Tang J. (2020). Trends in clinical development for PD-1/PD-L1 inhibitors. Nat. Rev. Drug Discov..

[150] Jiang Y., Zhao X., Fu J., Wang H. (2020). Progress and challenges in precise treatment of tumors with PD-1/PD-L1 blockade. Front. Immunol..

[151] Wu Y., Gu W., Li J., Chen C., Xu Z.P. (2019). Silencing PD-1 and PD-L1 with nanoparticle-delivered small interfering RNA increases cytotoxicity of tumor-infiltrating lymphocytes. Nanomedicine (Lond.).

[152] Li X., Wang R., Fan P., Yao X., Qin L., Peng Y., Ma M., Asley N., Chang X., Feng Y. (2019). A comprehensive analysis of key immune checkpoint receptors on tumor-infiltrating T cells from multiple types of cancer. Front. Oncol..

[153] Meng Y., Liang H., Hu J., Liu S., Hao X., Wong M.S.K., Li X., Hu L. (2018). PD-L1 expression correlates with tumor infiltrating lymphocytes and response to neoadjuvant chemotherapy in cervical cancer. J. Cancer.

[154] Feng M., Xu L., He Y., Sun L., Zhang Y., Wang W. (2018). Clinical significance of PD-L1 (CD274) enhanced expression in cervical squamous cell carcinoma. Int. J. Clin. Exp. Pathol..

[155] Chen H., Xia B., Zheng T., Lou G. (2020). Immunoscore system combining CD8 and PD-1/PD-L1: A novel approach that predicts the clinical outcomes for cervical cancer. Int. J. Biol. Markers.

[156] Feng Y.C., Ji W.L., Yue N., Huang Y.C., Ma X.M. (2018). The relationship between the PD-1/PD-L1 pathway and DNA mismatch repair in cervical cancer and its clinical significance. Cancer Manag. Res..

[157] Grochot R., Neto F.R., Tregnago A., Silva S., Scholze C., Norris R., Weschenfelder D., Pasqualotto F., Michelim L., Reiriz A.B. (2018). Clinical relevance of PD-L1 expression and its relation to tumor-infiltrating lymphocytes in cervical cancer. J. Clin. Oncol..

[158] Gadducci A., Guerrieri M.E. (2017). Immune checkpoint inhibitors in gynecological cancers: Update of literature and perspectives of clinical research. Anticancer Res..

[159] Sheu B.C., Chiou S.H., Lin H.H., Chow S.N., Huang S.C., Ho H.N., Hsu S.M. (2005). Up-regulation of inhibitory natural killer receptors CD94/NKG2A with suppressed intracellular perforin expression of tumor-infiltrating CD8^+^ T lymphocytes in human cervical carcinoma. Cancer Res..

[160] Hu Y., Liu T., Li J., Mai F., Li J., Chen Y., Jing Y., Dong X., Lin L., He J. (2019). Selenium nanoparticles as new strategy to potentiate γδ T cell anti-tumor cytotoxicity through upregulation of tubulin-α acetylation. Biomaterials.

[161] Jansen C.S., Prokhnevska N., Master V.A., Sanda M.G., Carlisle J.W., Bilen M.A., Cardenas M., Wilkinson S., Lake R., Sowalsky A.G. (2019). An intra-tumoral niche maintains and differentiates stem-like CD8 T cells. Nature.

[162] Verma V., Jafarzadeh N., Boi S., Kundu S., Jiang Z., Fan Y., Lopez J., Nandre R., Zeng P., Alolaqi F. (2021). MEK inhibition reprograms CD8^+^ T lymphocytes into memory stem cells with potent antitumor effects. Nat. Immunol..

[163] Algazi A.P., Twitty C.G., Tsai K.K., Le M., Pierce R., Browning E., Hermiz R., Canton D.A., Bannavong D., Oglesby A. (2020). Phase II trial of IL-12 plasmid transfection and PD-1 blockade in immunologically quiescent melanoma. Clin. Cancer Res..

[164] Abd Hamid M., Wang R.Z., Yao X., Fan P., Li X., Chang X.M., Feng Y., Jones S., Maldonado-Perez D., Waugh C. (2019). Enriched HLA-E and CD94/NKG2A interaction limits antitumor CD8^+^ tumor-infiltrating t lymphocyte responses. Cancer Immunol. Res..

